# Neurophysiological dynamics related to perception, action, and language in schizophrenia

**DOI:** 10.1126/sciadv.aeh5819

**Published:** 2026-10-21

**Authors:** Hsi T. Wei, Dominic Boutet, Rukun Dou, Jessica Ahrens, Nadia Zeramdini, Alban Voppel, Fernando Gonzales-Aste, Fatme Abboud, Sylvain Baillet, Lena Palaniyappan

**Affiliations:** ^1^ Department of Psychiatry, McGill University, Montreal, QC, Canada.; ^2^ Douglas Research Centre, McGill University, Montreal, QC, Canada.; ^3^ Montreal Neurological Institute, McGill University, Montreal, QC, Canada.; ^4^ Faculty of Medicine, McGill University, Montreal, QC, Canada.; ^5^ Integrated Program in Neuroscience, McGill University, Montreal, QC, Canada.; ^6^ Centre de recherche du Centre hospitalier de l’Université de Montréal.; ^7^ Department of Neuroscience, University of Montreal, Montreal, QC, Canada.

## Abstract

Schizophrenia disrupts both how people perceive the world and how they express and act on their thoughts, yet these symptoms are usually studied separately. Using magnetoencephalography during multisensory and motor tasks, we tested whether reduced event-related beta-band (15 to 30 hertz) modulation, a signal implicated in predictive control, provides a candidate neural marker across these domains. Compared to healthy individuals, patients showed diminished beta changes during sensory processing and movement completion, suggesting reduced flexibility in updating internal models. This neural pattern covaried with audiovisual simultaneity-judgment behavior, disorganized natural speech, and greater clinical severity. These findings suggest that distributed beta modulation may serve as a candidate cross-domain marker associated with perceptual, motor, and language disturbances in schizophrenia, motivating future studies that directly test predictive updating mechanisms.

## INTRODUCTION

A person with schizophrenia describes that the actors in a Netflix show produce “fake speech,” but their real voices come from the radiator vent in his room. When describing this experience, his speech appears disconnected with many ideas weakly developed—he moves from the Netflix “TV serial” to having “cereal” for “fastbreak” to “the vent making the room dry.” What links these seemingly disparate symptoms: one perceptual, one expressive? Both perception and language production depend fundamentally on the brain’s ability to generate predictions, compare them against incoming evidence, and flexibly update internal models. Within this predictive processing framework, beta oscillations (15 to 30 Hz) are a key converging measure of interest that have been proposed to support the maintenance and context-dependent updating of top-down predictive models, including cross-modal integration across the cortex (*1*–*3*). Here, we show that attenuated modulation of beta-band oscillations, a signal implicated in predictive control of internal models, may provide a candidate account of both perceptual aberrations and expressive symptoms in psychosis.

Multisensory integration (MSI) allows the brain to combine information from different sensory modalities to form a unified perceptual experience. This process is particularly critical when sensory inputs arrive at different times, requiring the brain to determine whether asynchronous signals originate from the same event. The presence of a temporal binding window [a time interval within which stimuli from different modalities are perceived as synchronous (*4*)] reflects the brain’s tendency to ascribe common cause to events a priori [i.e., internal predictive model of sensory world (*5*)]. This window is increased in schizophrenia and associated with perceptual disturbances (*6*) and speech processing (*7*), and in autism in relation to communication (speech production) deficits (*6*, *8*). A widened temporal binding window indicates an overreliance on the internal model of common cause (*5*) that is impervious to revisions (*9*, *10*). Alternative accounts, including increased sensory noise, attentional differences, or decision-level biases, could also contribute to broader binding windows in schizophrenia (*4*, *11*, *12*). Nonetheless, in the present study, the interpretation of reduced predictive flexibility is motivated not by the behavioral effect alone, but by its covariation with event-locked beta modulation as a flexibility index for internal models.

We adopt a specific and constrained interpretation of beta-band dynamics, focusing on event-related beta modulation as a candidate marker for studying predictive-updating flexibility across perceptual and expressive domains in psychosis. Under this formulation, attenuated beta modulation reflects reduced context-dependent manipulation of an ongoing predictive state, consistent with an insufficiently updated internal model. Alternative formulations, such as beta indexing top-down predictions or precision-weighting, are treated here as compatible computational accounts rather than distinct mechanisms directly adjudicated by the present data. In schizophrenia, predictive processing accounts propose aberrant precision weighting between prior beliefs and sensory evidence, resulting in internal models that may be insufficiently updated or overly constrained depending on context and hierarchical level (*13*–*17*). Neural processes underlying reduced flexibility in internal prediction models may contribute to both disrupted MSI and speech production abnormalities in schizophrenia.

Prior work implicates the inferior frontal gyrus (IFG) as a candidate higher-order region involved in audiovisual timing, action monitoring, and language control. Neuroimaging studies of audiovisual timing judgments and asynchrony detection implicate a right-lateralized fronto-insular and prefrontal network that includes the right IFG, suggesting that this region contributes to evaluating and updating perceived audiovisual timing (*18*–*20*). Neurophysiological evidence further indicates that neural activity in IFGs reflects supramodal, postsensory processes related to temporal prediction, integration, and decision-making, typically emerging several hundred milliseconds after stimulus onset rather than during early sensory encoding (*21*, *22*). Meanwhile, converging evidence from lesion, functional magnetic resonance imaging (fMRI), and electrophysiological studies indicates that the right IFG acts as a key control node within a fronto-basal-ganglia network supporting response inhibition and action cancellation, particularly when actions must be withheld or updated based on changing sensory information (*22*–*26*). IFG-mediated control is recruited when incoming sensory evidence violates expectations or signals the need to interrupt an ongoing or planned motor response. As such, IFG sits at the interface between sensory evaluation and motor output, enabling flexible adjustment of behavior based on temporally structured multisensory input.

Echoing the right IFG, left IFG involvement is more consistently observed in multisensory tasks that emphasize linguistic, semantic, or categorical processing demands, such as audiovisual speech perception or conflict resolution between competing interpretations (*27*–*31*). In particular, the left IFG is also critical for speech production, supporting the controlled selection (*32*–*35*), hierarchical sequencing, and predictive planning of linguistic and motor representations (*36*–*39*). The left IFG also plays a critical role in the two characteristic speech production abnormalities in schizophrenia: reduced lexico-semantic diversity (*40*, *41*) and syntactic complexity (*42*–*45*). Together, these literatures motivate the inclusion of bilateral IFG as a priori candidate regions when testing whether beta modulation across sensory, motor, and language-relevant systems covaries with perceptual and expressive abnormalities in schizophrenia.

Event-related beta modulation provides a candidate neurophysiological measure for testing this cross-domain pattern. Beta oscillations have been implicated in both sensory and motor processing, with a distinct role in posttask cortical responses. Poststimulus beta suppression reflects cortical engagement and processing of incoming sensory information (*46*–*48*) via reconfiguration of sensory predictions (*39*, *49*, *50*), whereas postmovement beta rebound (PMBR) signals the termination of motor activity and the stabilization of motor predictions (*46*, *49*, *51*, *52*) following the somatosensory feedback from action execution (*49*, *51*, *53*). These posttask responses in the audio/visual/motor cortices represent critical windows for updating or consolidating the internal models of the sensory/motor environment. This context-dependent beta modulation may reflect a distributed, system-level mechanism supporting the maintenance, updating, and coordination of internal models across cortex (*49*, *53*), which are crucial for complex acts such as speech production that demands precise temporal coordination of sensory feedback and motor output (*54*, *55*). Identifying latent patterns of coordinated task-related beta dynamics across sensory cortices (V1 and A1), motor cortex (M1), and IFG enables the study of its distributed system-level disruption in schizophrenia.

Here, we test the hypothesis that schizophrenia is characterized by a failure of context-dependent beta modulation, reflecting reduced flexibility in updating internal predictive models, and that this candidate deficit manifests across MSI, motor control, language organization, and clinical symptom expression.

## RESULTS

### Patients showed altered audiovisual simultaneity judgment

Auditory and visual stimuli were presented at different stimulus onset asynchronies (SOAs; see Materials and Methods). A relative measure of the likelihood to bind perception of obviously asynchronous stimuli was measured as the ratios of average synchronous response rate (SRR) in obvious (e.g., 750 and 1000 ms SOAs) versus ambiguous (e.g., 100, 150, and 200 ms SOAs) conditions (see section S2 for model-based psychophysical analysis). SRR ratios were computed separately for trials preceded by auditory-first (AF) and trials preceded by visual-first (VF) trial contexts and also after pooling across AF and VF trials.

SRR ratios (positively skewed; see section S1) pooled across AF and VF conditions were higher in patients than controls (*U* = 396, *P* = 0.018, rank-biserial *r* = 0.318; see section S1 for distribution diagnostics). Thus, patients showed a higher tendency than controls to report synchronous perception for obviously asynchronous stimuli. The pooled test was the prespecified primary comparison and was not included in the condition-specific false discovery rate (FDR) family. When trials were separated by AF and VF conditions, the same pattern was strongest for AF (*U* = 397.5, *P* = 0.007, *q*FDR = 0.014, rank-biserial *r* = 0.323) and trending for VF (*U* = 360, *P* = 0.10, *q*FDR = 0.10, rank-biserial *r* = 0.212). Some patients reported synchronous perception even for obviously asynchronous AF trials, resulting in SRR ratios greater than 1. Because SRR is defined as the ratio of synchronous responses for obvious versus ambiguous SOAs, values greater than 1 indicate that clearly asynchronous stimuli were judged synchronous as often as, or more often than, ambiguous stimuli. This pattern may reflect an expression of reduced sensitivity to detect asynchrony and a tendency to judge asynchronous events as simultaneous over “longer intervals,” consistent with prior schizophrenia simultaneity-judgment findings (*5*, *56*), with substantial yet genuine intersubject variability (*57*). In the present sample, three patients showed SRR ratio > 1 in at least one condition. Only one patient showed this pattern in both AF and VF conditions, whereas the other two showed condition-specific elevations and also occupied high values on independently derived clinical and semantic PCs measured outside the magnetoencephalography (MEG) task (section S1.3), suggesting that these values may include clinically meaningful high-end cases and making a purely random-response explanation less compelling. A sensitivity analysis using SRRs for the obviously asynchronous SOAs alone, without normalizing by ambiguous-SOA response rates, showed the same pattern, with a stronger auditory condition effect and a weaker visual condition effect (see section S1).

Nevertheless, because SRR ratios greater than 1 are psychophysically atypical and may raise response-validity or high-leverage concerns, we conducted a conservative sensitivity analysis excluding participants with pooled SRR ratio > 1, applying the same criterion to patients and controls. Two patients and no controls met this criterion. After these exclusions, the pooled group effect was attenuated (*U* = 346.0, *P* = 0.050), whereas the AF-specific effect remained significant (*U* = 347.5, *P* = 0.022). A denominator-free, obvious SOA–only analysis showed a similar direction of effect, although some results were attenuated after excluding SRR > 1 (see section S1.2). We therefore retain the full-sample SRR analysis as the primary test of overbinding at long SOAs, while treating the downstream SRR-dependent associations as requiring replication in larger samples. Together, these behavioral results indicate an increased tendency among patients to judge highly asynchronous audiovisual events as synchronous, consistent with prior reports of reduced asynchrony detection and widened audiovisual simultaneity windows in schizophrenia (*6*).

### Patients showed delayed and weaker motor and sensory-related beta modulation

Mean event-related beta power was extracted from the hypothesis-driven time windows defined in Table 1 and compared between patients and controls. Multiple-comparison correction was applied using FDR within the conceptual subfamilies defined by region of interest (ROI) class and time window (Table 1 notes).

**Table 1.** Event-related time windows motivated for beta power extraction. The table assigns distinct functional constructs to movement-locked beta: M1 and IFG within each time window, reflecting different theoretical roles (sensorimotor updating versus frontal cognitive control, motor preparation versus action selection, etc.). This gives us six conceptually distinct subfamilies (ROI type × window), each containing four tests (left/right hemisphere × left/right hand). Similarly, each audio- and visual-locked beta has two conceptual subfamilies, with each conceptual subfamily containing two tests (left/right hemispheres).

Audio-locked	A1	0–0.20	Early auditory-locked beta suppression following auditory onset; window motivated by auditory cortical response timing	(*87*–*89*)
	IFG	0.20–0.60	Supramodal postsensory beta modulation related to temporal prediction, multisensory integration, and perceptual decision-making, while minimizing contamination from motor preparation	(*90*, *91*)
Visual-locked	V1	0–0.20	Early visual-locked beta suppression following visual onset; window motivated by magnocellular visual pathway latencies	(*21*, *22*, *39*, *92*)
	IFG	0.20–0.60	Supramodal postsensory beta modulation related to temporal evaluation and decision processes, independent of sensory modality, while minimizing contamination from motor preparation	(*21*, *22*, *39*, *93*)
Movement-locked	M1	−0.50 to 0	Premovement beta desynchronization reflecting motor preparation and release from inhibition	(*46*, *94*, *95*)
	IFG	−0.50 to 0	Premovement frontal beta modulation related to action selection, response preparation, and top-down control	(*39*, *96*)
	M1	0–0.50	Early postmovement beta suppression/desynchronization reflecting movement execution and immediate postexecution sensorimotor processing	(*46*, *94*, *95*)
	IFG	0–0.50	Early postmovement frontal beta modulation related to action execution, response commitment, and early postdecision processing	(*39*, *96*)
	M1	0.50–1.0	Postmovement beta rebound (PMBR) associated with movement termination, sensory reafference, and motor state updating	(*46*, *94*, *95*)
	IFG	0.50–1.0	Postaction frontal beta related to cognitive control, monitoring, and response evaluation	(*39*, *96*)

Patients showed two abnormalities in movement-related beta modulation: (i) reduced early postmovement beta modulation in the left IFG for both left- and right-hand responses [left hand: Welch’s *t*(36.85) = −2.47, *P* = 0.018, *q*FDR = 0.040, Hedges’ *g* = −0.68; right hand: Welch’s *t*(34.15) = −2.47, *P* = 0.019, *q*FDR = 0.040, Hedges’ *g* = −0.68; Fig. 1A] and (ii) reduced M1 PMBR amplitude [LM1 left hand: Welch’s *t*(45.06) = −2.68, *P* = 0.010, *q*FDR = 0.022, Hedges’ *g* = −0.75; LM1 right hand: Welch’s *t*(46.00) = −2.35, *P* = 0.023, *q*FDR = 0.031, Hedges’ *g* = −0.67; RM1 left hand: Welch’s *t*(45.97) = −3.22, *P* = 0.002, *q*FDR = 0.010, Hedges’ *g* = −0.91; Fig. 1B]. Cluster-based time-frequency analyses further showed significant motor-locked beta clusters overlapping the predefined movement-related windows in left IFG, right M1, and left M1 (section S3).

**Fig. 1.**
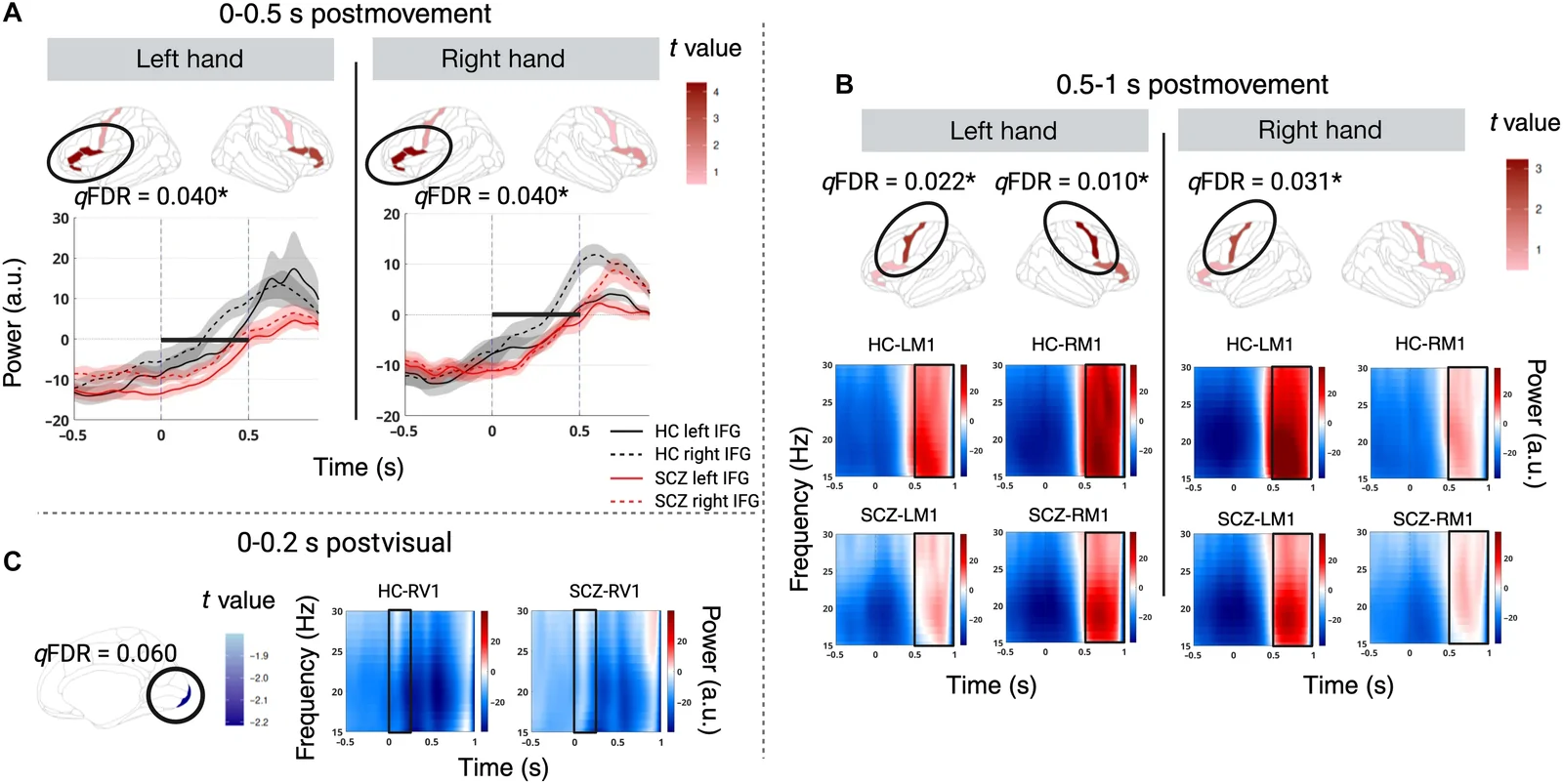
Postevent beta power difference between patients and controls. Patients with schizophrenia (SCZ; *N* = 23) versus healthy controls (HC; *N* = 25). In all brain maps, color shows the t value for mean beta power (15 to 30 Hz), computed as controls minus patients (opposite in sign to the t values reported in the text). Circled regions differed significantly after FDR correction. (**A**) Early postmovement window (0 to 0.5 s), shown separately for left- and right-hand button presses. Top: t values for M1 and IFG. Bottom: mean movement-locked beta power (±SEM, shaded) in left and right IFG (movement onset = 0 s). Gray dashed vertical lines mark 0 and 0.5 s, and the thick black horizontal bar marks the 0-to-0.5-s analysis window, drawn at baseline (0 power). Patients had lower left IFG beta power across this window, and their beta power rose above baseline later than in controls. (**B**) Postmovement beta rebound window (0.5 to 1 s). Top: t values for M1 and IFG. Bottom: movement-locked time-frequency maps for HC (upper row) and SCZ (lower row) in LM1 and RM1. Black boxes mark the analysis window. Patients showed weaker beta rebound in bilateral M1 for left-hand presses and in left M1 for right-hand presses. (**C**) Early postvisual window (0 to 0.2 s). Left: t value for RV1. Right: visual-locked time-frequency maps for HC and SCZ, with black boxes marking the analysis window. Patients showed a trend toward weaker early beta suppression in RV1 that did not survive FDR correction (qFDR = 0.060). “*” denotes qFDR < 0.05.

To assess whether PMBR effects could be explained by slower or more variable responses, we repeated the PMBR analyses while controlling for mean reaction time and RT coefficient of variation (PMBR ∼ group + mean RT + RT coef. of variance). Group effects remained significant after FDR correction within the M1 PMBR subfamily for LM1 left-hand PMBR (*B* = −10.31, standardized β = −0.69, partial *R*^2^ = 0.121, *P* = 0.019, *q*FDR = 0.025), LM1 right-hand PMBR (*B* = −20.00, standardized β = −0.82, partial *R*^2^ = 0.176, *P* = 0.004, *q*FDR = 0.010), and RM1 left-hand PMBR (*B* = −19.04, standardized β = −0.84, partial *R*^2^ = 0.170, *P* = 0.005, *q*FDR = 0.010). These results suggest that PMBR group differences are not explained by RT or RT variability.

For sensory-locked processing, the predefined scalar analysis showed nominal attenuation of early beta suppression in right V1, but this did not survive FDR correction within the V1 early visual subfamily [Welch’s *t*(41.61) = 2.21, *P* = 0.032, *q*FDR = 0.060, Hedges’ *g* = 0.64; Fig. 1C]. The corresponding left V1 effect was not significant [Welch’s *t*(30.35) = 1.80, *P* = 0.082, *q*FDR = 0.082, Hedges’ *g* = 0.53]. Early auditory-window tests in A1 were also not significant [LA1: Welch’s *t*(45.97) = 1.26, *P* = 0.215, *q*FDR = 0.353, Hedges’ *g* = 0.36; RA1: Welch’s *t*(41.95) = 0.94, *P* = 0.353, *q*FDR = 0.353, Hedges’ *g* = 0.27]. However, cluster-based time-frequency analyses showed significant beta-band clusters in right V1 and right A1 (section S3). The visual cluster overlapped the a priori early sensory window, whereas the auditory cluster emerged later, suggesting that auditory beta abnormalities may be more prominent during sustained postsensory evaluation than during initial auditory encoding. Baseline-control analyses indicated that these sensory-locked effects were unlikely to reflect group differences in baseline beta power (section S4).

Together, these findings indicate reduced event-related beta modulation in schizophrenia, particularly during early postmovement IFG modulation, M1 postmovement rebound, and sensory/postsensory beta dynamics. Within a predictive-processing framework, this pattern is consistent with impaired updating and stabilization of internal models: Attenuated beta suppression may reflect weaker incorporation of sensory evidence, whereas reduced PMBR may reflect less efficient postaction updating following movement-related feedback. Exploratory beta-band PTE analyses are reported in section S10. Because PTE was estimated over broad 0-to-1–s postevent windows to ensure sufficient beta cycles, these analyses were interpreted as directed statistical dependence evidence rather than temporally specific evidence for top-down control.

### Audiovisual simultaneity judgment relates to weaker event-related beta modulation

To investigate whether beta’s role in predictive model flexibility is supported by the association with multisensory binding behavior, behavioral partial least squares correlation (PLSC) was implemented with permutation testing for latent component significance and bootstrap resampling for feature reliability (see Materials and Methods). We identified a single significant latent component linking beta-band activity across distributed cortical regions (cf. Table 1) to behavioral measures of audiovisual simultaneity [latent variable (LV) 1: permutation *P* < 0.01; *n* = 5000 permutations]. This latent component revealed a coherent multivariate association between SRR ratio performance and beta-band dynamics spanning primary motor cortex, IFG, and early sensory cortices (Fig. 2). Bootstrap ratios (BSR) identified a beta pattern characterized by weaker premovement beta suppression in motor and IFG regions, reduced postsensory beta suppression in auditory and visual cortices and IFG, and attenuated PMBR in motor cortex (|BSR| > 1.96, 95% confidence; 5000 bootstraps). Across participants, stronger SRR ratio, reflecting a greater tendency to bind asynchronous sensory inputs, was associated with reduced modulation of beta activity during both sensory processing and movement termination, as reflected in the association between brain and behavioral latent scores [*r*(44) = 0.42, *P* = 0.0036]. Thus, greater synchrony judgments for asynchronous audiovisual events were associated with reduced beta modulation during sensory processing and movement termination, consistent with impaired updating of internal sensory-motor predictions. This behavior-beta association was stable in leave-one-participant-out (LOO) sensitivity analyses: the LV1 association remained in the same direction in 46 of 46 iterations, including iterations in which each participant with SRR > 1 was omitted, and the beta-feature weight pattern remained highly similar to the full-sample solution across iterations (LOO-to-full pattern correlation range: *r* = 0.974 to 1.000; see section S5). Thus, the full-sample association was not driven by any single participant, including those with SRR > 1. A complementary sensitivity analysis excluding both participants with pooled SRR ratio > 1 simultaneously preserved a positive latent-score correlation, although model-level permutation evidence was attenuated (see section S1.2). The association also remained significant after residualizing both beta and behavioral variables for diagnostic group (LV1: permutation *P* = 0.019; latent-score *r* = 0.388), indicating that it was not explained solely by mean patient-control differences (see section S6).

**Fig. 2.**
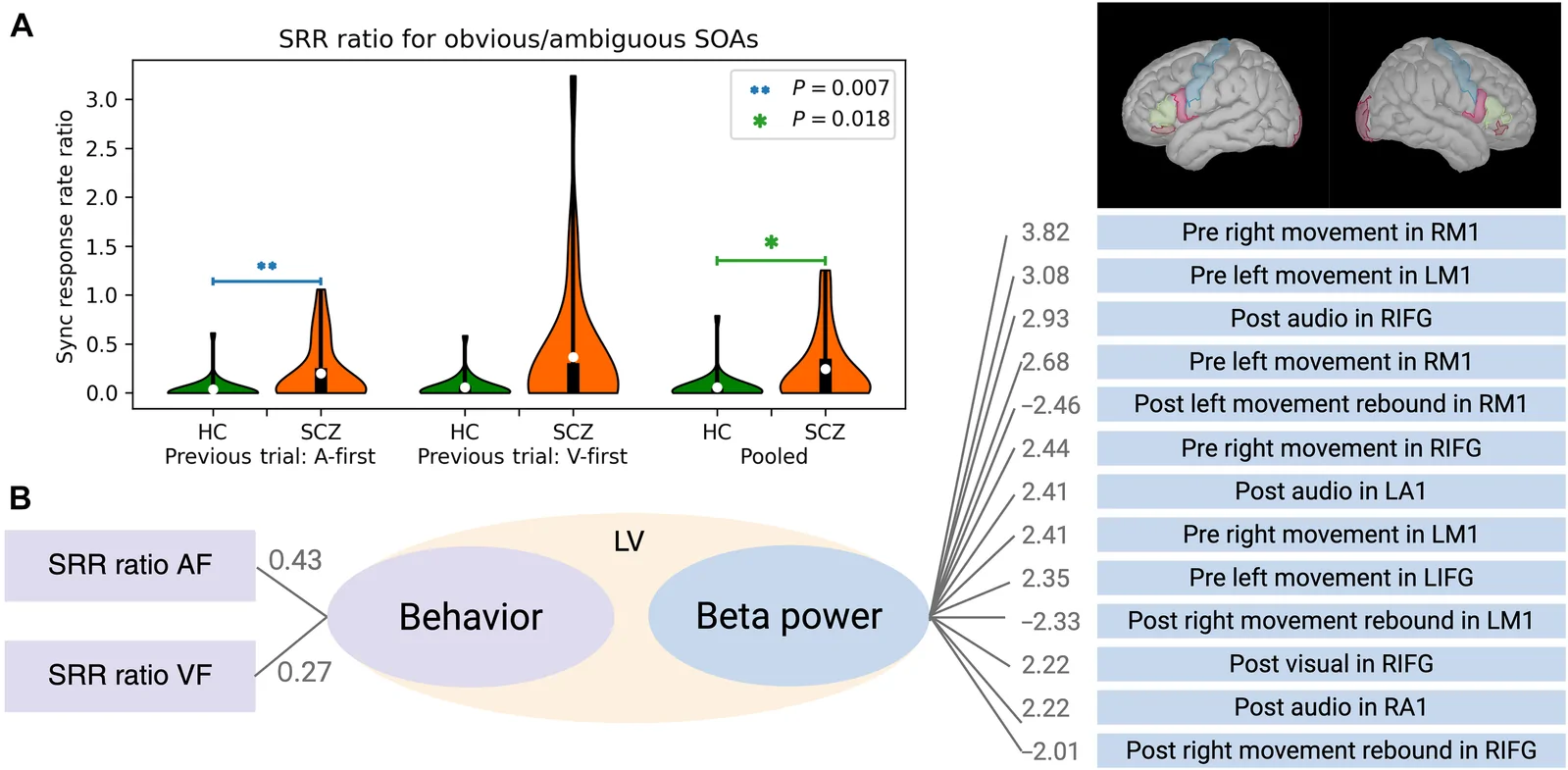
Latent association between beta-band activity and audiovisual simultaneity behavior. Behavioral PLSC identified a single latent component linking beta-band power features with SRR ratio measures (*N* = 46). (**A**) Violin plots show Mann-Whitney *U* group differences in SRR ratios for obvious versus ambiguous stimulus onset asynchronies (SOAs), separated by either AF and VF by preceding trials, or all AF and VF trials pooled together (*P* values indicated). (**B**) Numbers adjacent to SRR measures represent Y loadings (correlations between each SRR ratio and the behavioral latent score), indicating their contribution to the latent behavioral pattern. Right: Beta-band features contributing reliably to the neural latent component are shown with bootstrap ratios (salience divided by bootstrap standard error); only features exceeding the bootstrap threshold are displayed. Contributing beta features span M1, IFG, and A1/V1 across pre- and postevent time windows, indicating a distributed beta-band pattern linking sensory-motor processing to audiovisual simultaneity behavior. Sensitivity analyses excluding participants with SRR ratios greater than 1 are reported in section S1. “*” denotes significance (no need for FDR correction) and “**” denotes significance that survives FDR correction.

### Language production abnormalities show exploratory associations with audiovisual simultaneity judgment

Natural speech production was recorded and transcribed (see Materials and Methods) to extract speech features indexing stable patterns of individual speech characteristics instead of moment-to-moment production dynamics (see table S1 for language features and table S2 for natural language processing/NLP models used). To capture patterns of language production abnormalities while managing the high dimensionality of NLP-derived features from natural speech, we performed separate principal components analyses (PCAs) on semantic and syntactic measures. A semantic component indicating less semantic diversity (i.e., more similar words and sentences with lower semantic density) explained 53.57% of semantic feature variance. A syntactic component indicating lower syntactic complexity (i.e., less complex clauses lacking syntactic depth) accounted for 75.41% of the total syntactic feature variance.

Patients showed semantic impoverishment [i.e., higher scores on the semantic component; PT mean = 2.10, HC mean = −1.93; Welch’s *t*(22.29) = 6.24, *P* < 0.0001, Hedges’ *g* = 1.85] and syntactic simplicity [PT mean = 0.84, HC mean = −0.77; Welch’s *t*(42.64) = 4.30, *P* < 0.0001, Hedges’ *g* = 1.23] compared to controls. Two trained raters, both blind to the participant groups, used the short-form of the Thought and Language Index (TLI) scale (*58*) to assess signs of formal thought disorder, which refers to disturbances in the organization, coherence, and productivity of speech, encompassing both language disorganization (e.g., loosening of associations and peculiar sentences) and impoverishment (e.g., poverty of speech) (*59*). NLP-derived semantic impoverishment best captured TLI items poverty of speech [*r*(46) = 0.57, *P* = 0.000026] and weakening of goal [*r*(46) = 0.46, *P* = 0.00099] while the syntactic simplicity best captured peculiar sentences used by the patients [*r*(46) = 0.44, *P* = 0.0019].

Exploratory Pearson correlations suggested that higher mean SRR was associated with poorer speech organization, including semantic impoverishment [SRR-semantic PC: *r*(21) = 0.47, *P* = 0.024, *q*FDR = 0.024] and syntactic simplicity [SRR-syntactic PC: *r*(21) = 0.54, *P* = 0.008, *q*FDR = 0.015], after removing observations with zero mean SRR. Percentage-bend correlations, which downweighted the 20% most median-deviant observations, were attenuated to approximately *r* ≈ 0.40 but retained the same direction. We therefore treat the SRR-language correlations as exploratory support for a possible relationship between multisensory binding and language organization, which is primarily examined by the multivariate PLS analyses linking both behavioral and language measures to distributed beta modulation.

### Attenuated event-related beta modulation covaries with language disturbances

Using behavioral PLSC with permutation testing and bootstrap resampling (see Materials and Methods), we examined correlations between a latent beta dynamic reflecting the flexibility of the internal model and the semantic and syntactic language components. For both analyses, permutation testing identified a single significant latent component capturing language-beta covariance (semantic LV1: permutation *P* < 0.05; syntactic LV1: permutation *P* < 0.05; *n* = 5000 permutations).

In the semantic-beta model (Fig. 3A), BSRs (|BSR| > 1.96) revealed that semantic impoverishment (i.e., higher word-level and sentential semantic similarity with less diversity) was associated with weaker PMBR in bilateral M1s, weaker postsensory beta suppression in LA1 and RV1, and weaker premovement beta suppression in bilateral M1s [beta and semantic latent scores, *r*(46) = −0.50, *P* = 0.00029]. In the syntactic-beta model (Fig. 3B), reliable beta features included reduced PMBR in bilateral M1s, decreased postvisual beta suppression in V1s, and weaker beta suppression during motor preparation, particularly in the right M1. Syntactic simplicity (shorter, less deeply embedded clauses) was likewise associated with reduced beta modulation spanning sensory and motor domains [*r*(46) = 0.47, *P* = 0.00079].

**Fig. 3.**
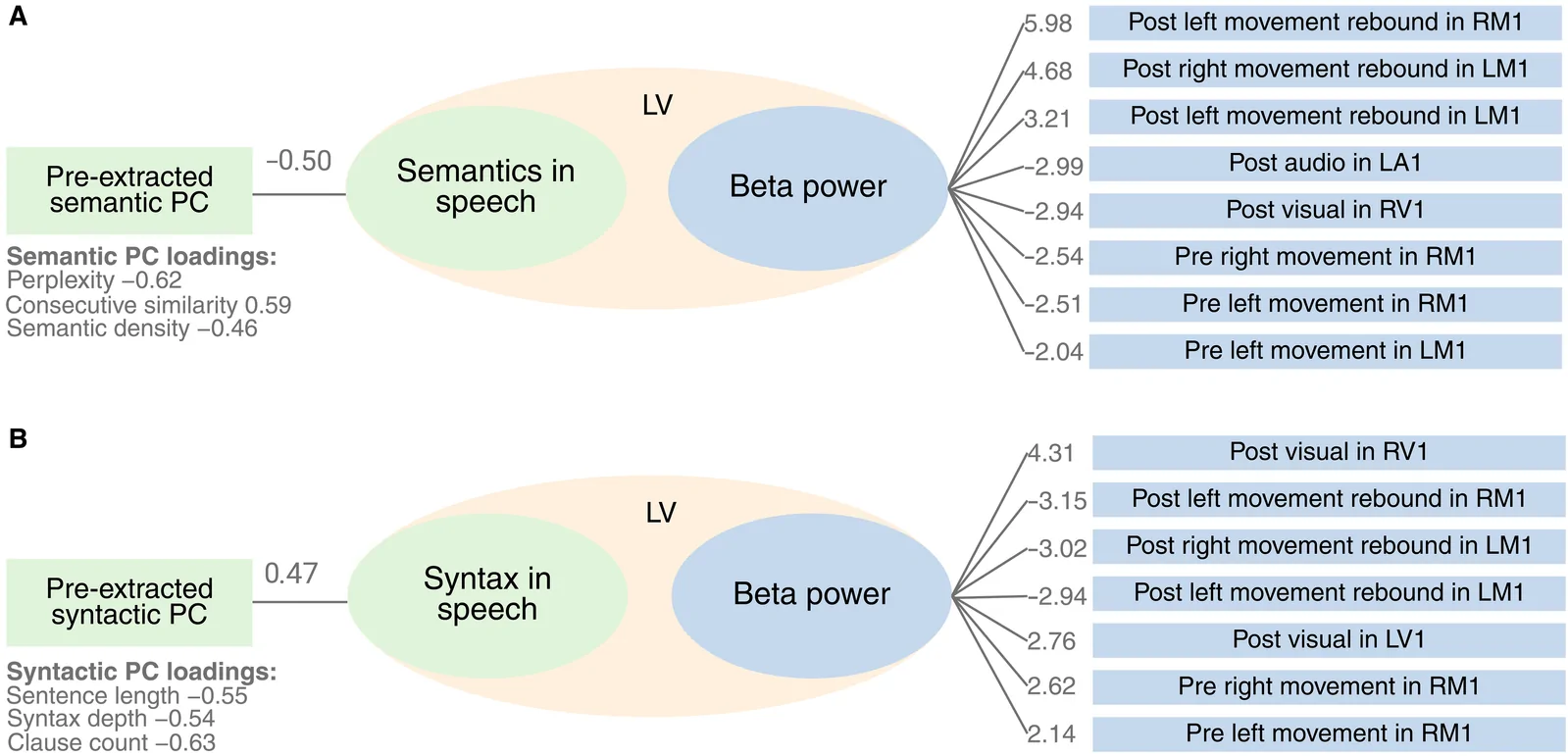
Latent associations between beta-band dynamics and pre-extracted semantic and syntactic language components. Behavioral PLS was used to relate beta-band power features to pre-extracted semantic (**A**) and syntactic (**B**) PCs derived from speech measures (*N* = 48). In each analysis, a single latent variable (LV) captured the shared covariance between beta activity and the corresponding language component. Left: Numbers adjacent to the pre-extracted PCs represent Y loadings (correlations between pre-extracted PCs and the LVs). PC loadings indicate the contribution of individual speech features to the semantic or syntactic component (only |loading| > 0.40 are shown). Right: Beta-band features contributing reliably to each neural latent pattern are shown, with values indicating bootstrap-normalized weights (bootstrap ratios); only features exceeding the bootstrap reliability threshold (|BSR| > 1.96) are displayed. For both semantic and syntactic models, reliable beta features were observed predominantly during premovement and postmovement intervals in primary motor cortex, as well as postsensory responses in auditory and visual cortices, indicating that beta dynamics during sensorimotor processing covary with higher-order linguistic organization.

Both language-beta associations were stable in LOO sensitivity analyses. The semantic PC-beta LV1 association remained in the same direction in 48 of 48 iterations, with highly stable beta-feature weight patterns across iterations (LOO-to-full pattern correlation range: *r* = 0.988 to 1.000). The syntactic PC-beta LV1 association also remained in the same direction in 48 of 48 iterations, with similarly stable beta-feature weight patterns (LOO-to-full pattern correlation range: *r* = 0.967 to 1.000; see section S5). The semantic and syntactic PCs were designed to summarize theoretically defined speech domains that showed prominent diagnostic differences; therefore, their covariance with beta modulation was expected to include group-related diagnostic variance. Accordingly, group-residualized sensitivity analyses attenuated the a priori semantic PC-beta and syntactic PC-beta models, which no longer reached significance after removing diagnostic-group effects from both beta features and speech PCs (semantic LV1: permutation *P* = 0.307; syntactic LV1: permutation *P* = 0.247; see section S6). These findings indicate that the primary speech-beta results reflect diagnosis-associated cross-domain covariance across the case-control sample, rather than diagnosis-independent individual-difference associations between beta modulation and speech organization.

We conducted spectral and spatial control analyses to clarify the specificity of these effects. Because gamma-band activity is commonly linked to feedforward sensory signaling and prediction-error processing (*39*, *60*, *61*) and prominently implicated in schizophrenia (*62*), we used gamma as a control frequency. Gamma-band control analyses showed one corrected group difference in LM1 gamma rebound [left hand: PT mean = −0.52, HC mean = 4.43; Welch’s *t*(37.38) = −3.05, *P* = 0.0042, *q*FDR = 0.017, Hedges’ *g* = −0.85], but gamma-band PLS models did not reproduce the primary behavior-, semantic-, or syntactic-beta associations. Control-ROI beta features extracted from bilateral medial orbitofrontal sulcus-olfactory cortex showed no event-related group differences (see sections S7 and S8).

Together, these results indicate that the disrupted beta dynamics across sensory and motor domains (i.e., weaker beta decrease during sensory processing and motor preparation and weaker PMBR), which also relate to audiovisual simultaneity-judgment behavior, covary with stable language organization features in schizophrenia. However, the SRR-dependent behavior-beta association was sensitive to exclusion of participants with pooled SRR ratios greater than 1 and requires replication in larger samples. Because speech was recorded outside the MEG session, these findings do not demonstrate beta abnormalities during language production itself. Rather, they indicate that individual differences in sensorimotor beta modulation are associated with stable language-organization features measured offline.

### Weaker beta modulation of internal model relates to worse psychotic symptoms in patients

With the smaller patient sample size (*n* = 23) that limits the permutation and bootstrapping methods, we undertook an exploratory assessment of whether the linguistic organization-related beta dynamics also related to clinical severity. One principal component was extracted from 10 clinical scores, explaining 58% of total variance in illness burden (see table S6). The illness burden was weighted by the core psychotic symptoms (e.g., higher positive and negative symptom severity and lower social functioning measures). Antipsychotic exposure [defined daily dose (DDD)] did not correlate with illness burden [*r*(21) = 0.18, *P* = 0.42]. Medication-sensitivity regressions further showed that antipsychotic exposure did not predict motor or sensory beta modulation, individual beta features after correction, or the behavior-, semantic-, or syntactic-beta latent scores after controlling for illness burden (see section S12).

The beta latent scores derived from the semantic-beta and syntactic-beta PLS models were correlated with illness burden: The latent beta dynamics related to semantic impoverishment (Fig. 3A) significantly correlated with illness burden [*r*(21) = −0.46, *P* = 0.027; weaker semantic-beta modulation seen with more severe burden] while syntactic simplicity related beta (Fig. 3B) had a trend level association [*r*(21) = 0.39, *P* = 0.06].

To clarify symptom specificity, we conducted exploratory patient-only correlations between positive- and negative-symptom components and the major behavioral, speech, and beta latent component measures (section S11). No correlation survived FDR correction. Direct SRR-clinical correlations were small, whereas the strongest nominal symptom-specific patterns linked positive/disorganization-spectrum symptoms with syntactic organization (Pearson *r* = 0.50, *P* = 0.014, *q*FDR = 0.308; Spearman ρ = 0.49, *P* = 0.018, *q*FDR = 0.281) and syntactic-beta latent scores (Pearson *r* = 0.45, *P* = 0.029, *q*FDR = 0.308; Spearman ρ = 0.45, *P* = 0.031, *q*FDR = 0.281).

Together, these findings suggest that beta modulation abnormalities in schizophrenia may emerge as a distributed latent pattern associated with multisensory processing, motor control, language organization, and clinical symptom expression.

## DISCUSSION

We provide preliminary evidence for attenuated beta modulation as a candidate cross-domain neural marker associated with perceptual, motor, and linguistic dysfunction in schizophrenia. To clarify the evidentiary weight of each finding, we organize this discussion around an explicit hierarchy: Primary evidence comprises case-control differences in event-related beta modulation and the beta-audiovisual association; secondary evidence comprises diagnosis-associated beta-speech covariance; and exploratory evidence comprises clinical associations and PTE analyses. This structure reflects differences in statistical directness, robustness checks performed, and a priori specification, not differences in scientific interest.

### Primary evidence: Case-control beta modulation and the beta-audiovisual association

Patients showed attenuated event-related beta modulation during sensory and motor processing, with the strongest univariate evidence in motor PMBR and additional support from cluster-based and multivariate analyses. These beta dynamics covaried with audiovisual simultaneity-judgment behavior, particularly the tendency to judge highly asynchronous audiovisual events as synchronous, supporting their interpretation as a candidate cross-domain marker for future validation.

A central contribution of this work is the specification of how predictive-processing abnormalities manifest in beta dynamics across distinct task contexts. Within predictive coding frameworks, psychosis has been characterized as reflecting either overly strong priors that dominate perception or weak priors that fail to stabilize inference (*14*–*16*). Our results refine these accounts by suggesting that a key abnormality may involve the dynamics of updating predictive models (indexed by event-related beta modulation) rather than the absolute strength of priors themselves, consistent with proposals that beta oscillations index the maintenance and precision-weighting of internal models (*1*, *2*, *39*). Under this formulation, elevated baseline or resting beta activity, as often observed in schizophrenia (*62*, *63*), can coexist with diminished event-related beta suppression or rebound, because the core deficit may lie in its context-sensitive regulation rather than oscillatory capacity itself [see (*64*) for a converging framework]. This distinction is important because our interpretation should not be taken to imply that beta-band activity is a uniform or disorder-specific spectral marker in schizophrenia [see heterogeneous resting-state EEG findings: (*65*)]. Rather, the present findings concern source-resolved, event-related beta modulation, which may capture regionally specific cortical dynamics that are less apparent in scalp EEG because MEG and EEG differ in source-orientation sensitivity and susceptibility to skull/scalp conductivity uncertainties (*66*–*68*). Thus, we interpret the observed beta effects as context-dependent and regionally specific, rather than as evidence for a global beta-band biomarker.

Consistent with this account of impaired context-sensitive beta regulation, we observed reduced beta suppression during sensory processing, alongside behavioral evidence of perceiving synchrony during obvious asynchrony. This pattern aligns with prior schizophrenia findings of reduced sensitivity to detect asynchrony and a tendency to judge asynchronous events as simultaneous over longer intervals (*5*, *56*). The temporal profile of this sensory beta abnormality differed between visual-locked and auditory-locked analyses. The visual-locked beta nominal effect was detected within the predefined early sensory window (0 to 0.2 s), whereas the auditory-locked beta effect emerged as a later cluster in the cluster-based permutation analysis, approximately 0.2 to 0.5 s after auditory onset. Thus, while visual beta disruption is expressed during initial sensory encoding, the auditory beta disruption emerges, somewhat unexpectedly, during sustained postsensory evaluation, but both are consistent with a shared disturbance in context-dependent predictive updating operating at modality-appropriate latencies (see section S3). As described above, transient beta suppression reflects a release of top-down constraints that permits sensory evidence to update perceptual predictions (*22*, *39*). Here, the reduced depth of beta suppression in patients is consistent with a less complete release of these top-down constraints, compatible with an internal model that is less readily updated (*13*, *16*). This effect was temporally specific and event locked, rather than a generalized reduction in beta power, supporting the interpretation of impaired modulation rather than diminished oscillatory capacity.

The same alteration in event-related modulation was expressed during motor behavior as delayed and attenuated PMBR. Consistent with the established role of PMBR in stabilizing predictive motor states (*46*, *49*, *53*), the delayed and weakened rebound observed in patients here suggests a failure to efficiently transition between predictive regimes following action [contrary to the model of imprecise priors (*69*)].

Within the predictive framework outlined above, the present findings constrain how failures of beta modulation manifest across sensory and motor domains. If beta supports the maintenance of internal models, then reduced flexibility in model updating (*13*, *14*) should manifest as diminished beta modulation following both sensory events that require updating perceptual predictions and motor feedback that signals the need to transition into a postmovement state. Together, these findings are consistent with the possibility that reduced predictive flexibility is expressed as distinct but related beta abnormalities across task contexts, without requiring multiple independent mechanisms.

This interpretation is also compatible with temporal-imprecision accounts of schizophrenia, which emphasize disturbances in the temporal organization of neural activity, including reduced signal-to-noise ratio, reduced intertrial phase coherence, phase instability, and phase-based temporal imprecision (*70*–*72*). Under this account, irregularities in ongoing neural phase dynamics may form a disrupted temporal “background” that compromises the precision with which task-relevant sensory and cognitive events are organized in the “foreground” (*73*). The present study did not directly test phase coherence, phase stability, or temporal precision indices, and therefore cannot adjudicate this model directly. However, the beta-power findings reported here are compatible with this framework. Event-related beta suppression and rebound require temporally coordinated transitions between predictive states; thus, reduced or delayed beta modulation may represent one power-based, event-locked expression of a broader temporal coordination deficit. From this perspective, phase-based temporal imprecision and attenuated beta-power modulation are complementary rather than competing explanations: Phase instability may constrain the timing precision needed for flexible updating, whereas reduced beta modulation may reflect a failure of task-relevant sensory and motor events to appropriately reconfigure ongoing predictive states. Future studies combining beta-power modulation with phase-based measures, such as intertrial phase coherence, phase stability, and beta-burst timing, will be needed to determine how temporal imprecision relates to impaired predictive updating in schizophrenia.

### Secondary evidence: Diagnosis-associated beta-speech covariance

These beta abnormalities extended beyond perception and action to natural language production, a domain that critically depends on predictive control. Although we did not measure beta activity during speech production itself, we observed that the same patterns of attenuated sensory and motor beta modulation covaried with reduced semantic diversity and syntactic complexity in spontaneous speech. This diagnosis-associated cross-domain association supports, but does not by itself establish, the interpretation that beta-mediated predictive mechanisms engaged during sensorimotor processing are also relevant for language generation. This interpretation aligns with converging evidence that beta oscillations support the maintenance and sequencing of linguistic context and the preactivation of lexical-syntactic representations during language processing, though not directly during language production (*38*, *42*, *43*). Under this account, insufficient beta modulation may reflect a failure to flexibly preactivate and update linguistic representations, biasing production toward high-probability, generic lexical items and simplified syntactic structures, as observed in formal thought disorder (*74*, *75*).

We flag this association as secondary rather than primary because the language-beta relationship was diagnosis dependent: After residualizing for diagnostic group, the a priori semantic PC-beta and syntactic PC-beta models no longer reached significance. These findings therefore support diagnosis-associated beta-speech covariance, but not diagnosis-independent beta-speech coupling at the individual-difference level.

### Exploratory evidence: IFG/PTE and clinical associations

The IFG findings should be interpreted cautiously. IFG showed temporal delays in beta rebound and contributed to the multisensory binding-related beta pattern, consistent with its proposed role in coordinating transitions between predictive states and evaluating supramodal timing. However, local IFG beta-power features were not consistently reliable contributors to the speech-related beta covariance models. Exploratory beta-band PTE analyses reported in section S10 suggested that IFG-linked directed statistical dependence covaried with sensory and motor beta modulation. However, PTE was estimated over broad 0-to-1 s postevent windows instead of the more fine-grained temporal windows in Table 1. We interpret IFG as an a priori candidate region within a broader sensory-motor beta network rather than direct evidence for temporally specific top-down control, although beta-band oscillations may carry top-down influences and support predictive coordination across cortical hierarchies (*76*, *77*).

The exploratory clinical analyses further clarify the level at which the present cross-domain association should be interpreted. Consistent with recent meta-analytic evidence that direct associations between audiovisual MSI measures and clinical symptoms in schizophrenia are typically small (*78*), we do not interpret behavioral MSI abnormalities alone as a strong clinical severity marker. Instead, the present findings suggest that multisensory binding, language organization, and clinical illness burden may be linked more strongly through distributed beta-related neural patterns than through a direct MSI-symptom relationship. This framing is also consistent with the symptom-specific exploratory analyses reported in section S11, where direct SRR-clinical correlations were small, while the strongest nominal patterns involved syntactic organization and syntactic-beta latent scores in relation to positive/disorganization-spectrum symptoms.

### Cross-cutting interpretive constraints

An important constraint on the present interpretation is that predictive-model flexibility was not directly manipulated or formally modeled. We interpret attenuated event-related beta modulation as an index of reduced flexibility in updating internal models based on prior accounts of beta oscillations in maintaining the status quo, reinstating internal representations, and regulating context-dependent updating. In the present data, this interpretation is indirectly supported by the convergence between reduced beta modulation, increased binding of asynchronous audiovisual events, and less flexible semantic and syntactic organization in natural speech. However, the study does not directly adjudicate between alternative computational accounts, such as altered precision weighting, reduced sensory reliability, attentional differences, or decision-level biases. Future work should directly manipulate prediction strength, sensory uncertainty, and update demands to test whether beta modulation tracks predictive updating flexibility in schizophrenia.

### Limitations

Limitations include the relatively small sample size, absence of an independent replication cohort, and cross-sectional design, which limit statistical power, mediation/causal inference, and generalizability. Although permutation testing, bootstrap resampling, and LOO analyses supported the stability of the reported PLS patterns, these internal checks do not substitute for external replication. Medication effects, while not supported by present sensitivity analyses, cannot be fully ruled out. Several design and measurement features warrant careful interpretation. The analyses were restricted to beta power rather than phase, temporal precision, beta bursts, or network coordination. Speech was recorded offline, so the present analyses reflect cross-domain trait-level covariation and not whether beta modulation directly supports online language processing or speech production. The NLP analysis focused on theory-driven semantic and syntactic features and did not comprehensively model broader linguistic or affective dimensions. The language-beta associations were also dependent on diagnostic-group structure: After residualizing for diagnostic group, the a priori semantic PC-beta and syntactic PC-beta models no longer reached significance. These findings therefore support diagnosis-associated beta-speech covariance, but not diagnosis-independent beta-speech coupling at the individual-difference level. The audiovisual task lacked auditory-only and visual-only control trials, so sensory-locked beta effects should be interpreted within an audiovisual context. The SRR-dependent associations were sensitive to SRR ratio > 1, which may represent clinically meaningful high-end cases rather than purely invalid behavioral observations. The findings speak to overall audiovisual simultaneity judgment tendency and overall beta modulation, not to SOA-specific neural mechanisms, the investigation of which will require a purpose-built parametric design. In addition, spatial leakage between nearby regions such as M1 and IFG cannot be fully excluded. Future larger, longitudinal, and independently replicated studies should test broader spatial and spectral patterns, examine first-episode or unmedicated patients, and directly measure oscillatory activity during online language processing and speech production.

In summary, by demonstrating that attenuated event-related beta modulation relates to aberrant MSI, disorganized speech, and clinical severity, this work provides important preliminary support for an account of perceptual and expressive dysfunction in psychosis, potentially reflecting reduced flexibility in updating internal models across perception and action. Because the present study was cross-sectional and correlational, future work should test whether beta modulation directly indexes predictive updating flexibility using experimental manipulations of prediction strength, sensory uncertainty, and update demands. By linking this marker to behavioral and linguistic disturbances, our findings position beta dynamics as a promising marker for future validation and as a potential target for mechanistically informed interventions [see (*79*) for a recent review on event-related beta as a neuromodulatory treatment target] aimed at restoring predictive flexibility in disorders of disrupted inference.

## MATERIALS AND METHODS

### Participants

This study followed a case-control design involving 25 patients with schizophrenia (age: 33.26 ± 9.03 years; 8 females) and 25 age- and sex-matched healthy controls (age: 33.36 ± 9.26 years; sex: 7 females). Patients and controls did not differ in age [Welch’s *t*(45.83) = −0.04, *P* = 0.970, Hedges’ *g* = −0.01], but patients had fewer years of education than controls [patients: *M* = 12.35 years, SD = 3.58; controls: *M* = 18.98 years, SD = 3.80; Welch’s *t*(45.98) = −6.23, *P* < 0.001, Hedges’ *g* = −1.76]. The study was approved by the Research Ethics Boards of McGill University and the Douglas Mental Health University Institute (REB: 2024-925), and all participants provided written informed consent in accordance with institutional guidelines. Inclusion criteria included English- or French-speaking participants, male or female, aged 18 to 65 years. Patients were recruited from the inpatient, outpatient, and community services caseload held by consultant psychiatrists working at McGill-affiliated hospitals in Montreal, Quebec. Patients had to be previously diagnosed with schizophrenia or schizoaffective disorder based on the *Diagnostic and Statistical Manual of Mental Disorders 5th Edition*. Consenting participants eligible for the study underwent symptom and speech assessment, as well as MEG and MRI recordings. Two patients were excluded from MEG analysis (MEG *N* = 48) due to dental and piercing-related artifacts. A different set of two patients (behavioral *N* = 48) were excluded from behavioral analysis due to inability to follow task instruction. When associating MEG with behavioral data, the sample size was 46.

Clinical assessments including SOFAS (*80*), PANSS-8 (*81*), and CGI-S (*82*) were implemented. A total of 10 numeric clinical rating scores were measured. To extract a single latent factor indicative of illness burden before associating clinical scores with MEG and speech metrics, PCA was used to extract the first component (explaining 58% of total variance) (see table S6). For each participant, a DDD was determined for psychotropic medications (antipsychotics, antidepressants, benzodiazepines, stimulants, anticonvulsants, beta blockers, and anticholinergic drugs separately). Participants provided a list of current medications, including maximum dose and duration. Where necessary, medication dosage was verified in their medical file. DDD per drug class was determined by ATC/DDD Index (https://atcddd.fhi.no/atc_ddd_index/), where the participant’s maximum prescribed daily dose was divided by the WHO DDD.

### MSI task

During MEG recording, participants completed an audiovisual simultaneity-judgment task. Each trial presented a white square (visual; V for 50 ms) at the screen center and a 1000-Hz tone (auditory; A for 50 ms) with one of 11 SOAs: 0, ±100, ±150, ±200, ±750, and ±1000 ms (negative values = audio first). Most SOAs were presented eight times per block for each lag direction (e.g., +100 ms and −100 ms each repeated eight times). The ±750- and ±1000-ms SOAs were presented twice per block and served only as “obviously asynchronous” anchors.

Visual stimuli were back-projected at 120 Hz, and tones were delivered binaurally via foam-tip earphones. On each trial, participants judged the pair as synchronous (S) or asynchronous (A) and responded using button pads positioned on both sides of the MEG chair. The S/A mapping to left/right buttons was pseudo-randomized within each block to counterbalance response laterality. After stimulus presentation, the letters “A” and “S” briefly appeared on the corresponding response sides to minimize rule-forgetting; they disappeared immediately upon button press. The task comprised five blocks of 64 randomized trials each (total: 320 trials). Blocks began when participants pressed the button assigned to the synchronous response. Intertrial intervals were jittered between 2.3 and 2.8 s, during which only a fixation cross was displayed. Two brief preparation blocks preceded the main task. The first familiarization block presented each SOA once in a fixed sweep order (−1000 to +1000 ms), with the SOA value shown beforehand to give participants an intuitive reference. The second was a short practice block presenting each SOA once in random order using the standard trial format.

### Behavioral analysis

For each participant, trials were pooled into conditions according to three properties: (i) the magnitude of SOA value in milliseconds; (ii) the sign of the SOA, which corresponds to the leading sensory modality of the stimulus (negative being auditory-first and positive being visual-first), and (iii) the leading sensory modality of the stimulus from the previous trial. For each condition, we computed an SRR, which corresponds to the percentage of trials in a given condition for which the participant reported perceiving the stimulus as synchronous. We categorized conditions with SOA of ±750 and ±1000 ms as “obvious” and those with SOA of ±100, ±150, and ±200 ms as “ambiguous.” We averaged the SRR for obvious and ambiguous conditions before computing the ratio between these average SRRs in AF (preceding trial is audio-first) and VF (preceding trial is visual-first) conditions separately and also when pooled across AF and VF trials. Note that two participants in the patient group were removed due to clear issues in their response patterns (e.g., giving the same response for entire blocks; *N* = 48). See section S2 for model-based psychophysical analysis on subjective simultaneity and temporal recalibration.

Participants with SRR ratio > 1 were retained in the primary analyses because these values may represent an extreme form of overbinding at long SOAs, but the SRR behavioral group comparisons and behavior-beta PLS analysis were repeated after conservative exclusion of participants with pooled SRR ratio > 1 to address the possibility that SRR ratio > 1 reflected psychophysically atypical response patterns, task disengagement, or response-mapping errors. These criteria were applied identically to patients and controls. These exclusions were used only as sensitivity analyses and did not replace the primary sample.

### Natural speech recording and analysis

Speech samples were collected using the Discourse in Psychosis Consortium protocol (*83*). The speech protocol took around 20 min of administration and consisted of seven sections, including free personal narrative, health narrative, picture description, storytelling, dream reports, reading, and recall. Audio recordings from participants’ interviews are transcribed to text using WhisperX (https://github.com/m-bain/whisperX) before manual correction of transcription and speaker diarization was done. All sentences from the interviewer were removed such that the textual data submitted to the analysis pipeline only contain utterances from the participant. The text associated with each participant was analyzed independently to generate a set of 30 speech features describing the language characteristics of the participant. The speech features extraction pipeline relies on both basic NLP algorithms and various types of Large Language Models (LLMs; see table S2). From our speech samples, semantic and syntactic features were extracted. Semantic features capture how meaning unfolds across discourse, using measures such as consecutive sentence similarity (average cosine similarity between sentence embeddings), lexical predictability (perplexity and pseudo-perplexity), and semantic density (the proportion of meaningful components within a sentence vector). Syntactic features index structural complexity based on part-of-speech parsing, including sentence length, syntactic tree depth, and clause count.

### MEG recording

MEG data were acquired using a whole-head 275-channel CTF MEG system (CTF/VSM, Coquitlam, Canada) housed in a magnetically shielded room at the Montreal Neurological Institute. Signals were recorded with axial gradiometers and online third-order synthetic gradiometer noise reduction, sampled at 1200 Hz. Participants were seated upright with head support to minimize movement. Head position was monitored continuously using three localization coils placed at the nasion and left/right preauricular points and relocalized at the start of each block. Individual head shapes (∼100 scalp points including fiducials) were digitized using a FASTRAK electromagnetic tracking system (Polhemus, USA) and used to coregister MEG data to each participant’s structural MRI via rigid-body transformation optimized by dense scalp surface alignment. Horizontal and vertical electrooculography (EOG) and electrocardiography (ECG) were recorded concurrently to aid artifact identification. Datasets with within-block head movement exceeding 5 mm or transient displacements >10 mm were flagged for review. Noisy or malfunctioning channels were identified using CTF system diagnostics and excluded before preprocessing.

### Structural MRI acquisition for MEG coregistration

T1-weighted images were collected from all participants with the 3T Siemens Prisma MRI scanner at the Neuro. The cortical surface used in MEG source modeling was derived using FreeSurfer 7.4.1 (recon-all) on a preprocessed skull-stripped image obtained by applying presurfer (https://github.com/srikash/presurfer) on the UNI and INV2 maps obtained from the MP2RAGE sequence. The resulting cortical surface was then imported into Brainstorm to perform, and refine, coregistration between the MRI and MEG data by aligning the Polhemus FASTRAK fiducials (75 to 100 headpoints including nasion and preauricular points) to their corresponding MRI landmarks, followed by refinement using the densely digitized scalp surface points. The resulting transformation matrix was used for forward model computation and subsequent source-space analyses.

### MEG preprocessing and time-frequency analysis

MEG preprocessing and source modeling were performed in Brainstorm using automated MATLAB scripts. Continuous CTF recordings and corresponding empty-room data were imported, realigned to digitized head points, and band-pass filtered (0.1 to 100 Hz). Auditory onsets were identified from the analog trigger channel. Cardiac and ocular artifacts were detected using peak-based methods on ECG and vertical EOG (VEOG) channels; signal-space projection (SSP) components were computed, manually reviewed, and applied. Cleaned data were epoched from −3 to 1 s relative to left/right button presses, auditory onsets, and visual onsets.

Noise covariance was estimated from empty-room recordings and data covariance from all trials. Forward models were constructed using overlapping spheres on individual MRIs, and source estimation used a linearly constrained minimum variance (LCMV) beamformer with neural activity index normalization and unconstrained dipoles. LCMV beamforming was used to improve spatial selectivity of source estimates (*84*, *85*), but no additional scout-level signal orthogonalization or leakage-correction procedure was applied between regions. Dipole orientations were reduced using PCA (first component) computed over a baseline window (−2.5 to −2.0 s). Source estimates were averaged across runs per participant and projected onto a standard cortical template.

ROIs were defined using the Destrieux atlas (*86*), including IFG subdivisions, primary motor cortex (precentral gyrus/M1), primary visual cortex (occipital pole/V1), and primary auditory cortex (transverse temporal cortex/A1), with all vertices included. Time-frequency decomposition was performed on source-level ROI signals using Morlet wavelets (1 to 60 Hz, 1-Hz steps), yielding baseline-normalized event-related spectral perturbations (ERSPs). Motor-related activity was baselined to −2.5 to −2.0 s to avoid movement preparation, while sensory-related activity was baselined to −1.5 to −1.0 s separately for auditory-first and visual-first trials to avoid stimulus contamination. These ERSP maps constituted the measures used for group-level analyses.

### Beta-band power extraction and time-window selection

To obtain participant-specific indices of beta-band modulation, for each participant, run-averaged ERSPs were computed for motor (left/right button press) and sensory (auditory and visual) events. Within anatomically defined ROIs, we computed the average ERSP map over all vertices. Average beta (15 to 30 Hz) powers were then extracted from each ROI’s ERSP map according to the event and time window specified in Table 1.

For sensory-locked analyses, auditory-locked windows were extracted from trials in which the auditory stimulus followed the visual stimulus, and visual-locked windows were extracted from trials in which the visual stimulus followed the auditory stimulus, and the baseline period always precedes the first stimulus; therefore, the postevent windows do not contain a subsequent stimulus onset, although all responses were measured within an audiovisual trial context. We focused on beta-band power modulation as a robust, interpretable first-order index of predictive control dynamics, noting that burst-based or phase-based analyses represent complementary levels of description beyond the scope of the present study.

### Statistical analysis

For group comparisons, independent-samples *t* tests were used when assumptions of homogeneity of variance were satisfied. When Levene’s test indicated unequal variances, Welch’s *t* tests were used, with degrees of freedom estimated using the Welch-Satterthwaite approximation. Effect sizes for parametric group comparisons were reported as Hedges’ *g*, and effect sizes for Mann-Whitney *U* tests were reported as rank-biserial correlations. For hypothesis-driven univariate group comparisons of beta power, multiple-comparison correction is applied within each subfamily as defined in Table 1, controlling the FDR within each conceptually independent hypothesis. Results that reached nominal significance but did not survive correction are reported as trend level. For exploratory time-frequency analyses not driven by specific hypotheses, we used cluster-based permutation testing to control family-wise error rate (see section S3).

To relate beta-band features to behavioral, speech, and clinical measures, we used behavioral PLSC as implemented in the pyls Python package. For each model, the *X* matrix contained subject-wise beta-power features and the *Y* matrix contained behavioral, speech, or clinical variables. All *X* and *Y* variables were *z*-scored across participants before analysis. PLSC decomposes cross-block covariance into LVs, with LV significance assessed using permutation testing (5000 permutations) and feature reliability assessed using bootstrap resampling (5000 bootstraps). Feature contributions with |BSR| > 1.96 were interpreted as reliable. Primary PLS analyses were conducted across all participants to identify disease-relevant cross-domain covariance. As a sensitivity analysis, we repeated the behavior-beta, semantic PC-beta, and syntactic PC-beta models after residualizing both beta features and behavioral/language variables for diagnostic group (see section S6).

To assess whether the reported PLS findings were driven by individual high-leverage observations, we conducted LOO robustness analyses for the three PLS models reported in the main text: audiovisual simultaneity behavior-beta, semantic PC-beta, and syntactic PC-beta. In each LOO iteration, one participant was removed, the remaining data were restandardized, and the PLS model was refit. Because PLS solutions can arbitrarily reverse sign, each LOO solution was sign aligned to the full-sample solution using the beta-feature weight vector. We then quantified robustness using (i) the LV1 latent-score correlation after each participant removal, (ii) the proportion of LOO iterations preserving the full-sample direction of association, (iii) the correlation between each LOO beta-feature weight vector and the full-sample beta-feature weight vector, and (iv) the overlap between the strongest beta-feature contributors in each LOO model and the full-sample solution. These analyses were used as internal sensitivity checks and were not considered a substitute for independent replication.

Pearson correlations were used to summarize associations between paired continuous measures, including associations between PLS-derived beta and behavioral/language latent scores, exploratory associations between multisensory binding and speech components, and patient-only associations between clinical illness burden and beta latent scores. As an outlier-robust sensitivity analysis for SRR-speech correlations, we computed percentage-bend correlations with β = 0.20. In the LOO PLS robustness analyses, both Pearson and Spearman latent-score correlations were recomputed across iterations to assess whether the reported associations were robust to removal of individual participants.

## Supplementary Materials

### This PDF file includes:

Supplementary Text Figs. S1 and S2 Tables S1 to S7 References
