# Supplementary material for: Neurophysiological dynamics related to perception, action, and language in schizophrenia
Source: Sci Adv. 2026 Oct 21;12(43):eaeh5819. doi: 10.1126/sciadv.aeh5819 (PMC13685960; doi:10.1126/sciadv.aeh5819)
Supplement: Supplementary file 1 [file sciadv.aeh5819_sm.pdf]

Science Advances
AAAS

Supplementary Materials for

Neurophysiological dynamics related to perception, action, and language in schizophrenia

Hsi T. Wei *et al.*

Corresponding author: Lena Palaniyappan, lena.palaniyappan@mcgill.ca

Sci. Adv. **12**, eaeh5819 (2026)
DOI: 10.1126/sciadv.aeh5819

This PDF file includes:

Supplementary Text
Figs. S1 and S2
Tables S1 to S7
References

Supplementary Text

1. Synchronous response rate (SRR) further analyses

1.1. SRR ratio distribution diagnostics

SRR ratio distributions departed from normality in both conditions, as indicated by significant Shapiro-Wilk tests and elevated skewness/kurtosis values. In the visual condition, distributions were positively skewed and leptokurtic for both HC, skewness = 3.41, kurtosis = 11.95, $W = 0.51$, $p < 0.001$, and SCZ, skewness = 2.76, kurtosis = 7.28, $W = 0.56$, $p < 0.001$. In the auditory condition, non-normality was also observed for HC, skewness = 4.11, kurtosis = 16.24, $W = 0.34$, $p < 0.001$, and SCZ, skewness = 1.51, kurtosis = 1.08, $W = 0.71$, $p < 0.001$. Therefore, Mann-Whitney U tests were used as the primary inferential tests for SRR ratios. SRR ratio did not significantly differ between groups in the visual condition ($U = 360.0$, $p = 0.101$, qFDR = 0.101), but differed significantly in the auditory condition ($U = 397.5$, $p = 0.007$, qFDR = 0.014), after FDR correction across the two condition-specific comparisons. Parametric t-tests showed a similar auditory effect, $t = 2.43$, $p = 0.019$, while the visual effect was only marginal, $t = 2.02$, $p = 0.049$, and was therefore interpreted cautiously.

As a sensitivity analysis addressing potential ratio bias, we also tested group differences in synchronous response rates for the obviously asynchronous SOAs alone, without normalizing by ambiguous-SOA response rates. This analysis avoids dividing estimates based on unequal trial counts. The pooled obvious-SOA-only SRR across auditory and visual conditions was higher in patients than controls ($U = 386.0$, $p = 0.032$). The pooled test was the pre-specified primary comparison and was not included in the direction-specific FDR family. The auditory condition effect remained significant after FDR correction across the two condition-specific comparisons ($U = 392.5$, $p = 0.010$, qFDR = 0.020), whereas the visual condition remained weaker and non-significant ($U = 360.5$, $p = 0.099$, qFDR = 0.099). Thus, the group difference in reporting synchrony for clearly asynchronous trials was not solely attributable to the obvious/ambiguous SRR-ratio calculation, although the ratio-based measure remains non-normal and is interpreted cautiously.

1.2. Interpretation and sensitivity analysis of SRR ratios greater than 1

The SRR ratio was designed to quantify the relative tendency to judge clearly asynchronous audiovisual events as synchronous compared with ambiguous events. Therefore, SRR values greater than 1 indicate an extreme response pattern in which obvious SOAs are judged synchronous as often as, or more often than, ambiguous SOAs. This pattern is not invalid by definition, because reduced sensitivity to detect asynchrony and judging asynchronous events as simultaneous over longer intervals are established features of schizophrenia simultaneity-judgment performance. We therefore retained participants with SRR ratio > 1 in the primary full-sample analyses and treated exclusions as conservative sensitivity analyses.

Nevertheless, because SRR ratios greater than 1 may also reflect psychophysically atypical response patterns, task disengagement, random responding, or response-mapping errors in some participants, we added conservative sensitivity analyses excluding these values. The exclusion criteria were applied identically to patients and controls. Two patients and no controls had pooled SRR ratios greater than 1.

After excluding participants with pooled SRR ratio > 1, the pooled SRR group difference was attenuated to trend level (patients: $N = 21$; controls: $N = 25$; $U = 346.0$, $p = 0.050$, rank-biserial $r = 0.318$, 95% CI [0.006, 0.613]). The AF-specific effect remained significant ($U = 347.5$, $p = 0.022$, qFDR = 0.044, rank-biserial $r = 0.324$, 95% CI [0.055, 0.585]), whereas the VF effect was non-significant ($U = 310.0$, $p = 0.246$, qFDR = 0.246, rank-biserial $r = 0.181$, 95% CI [-0.122, 0.482]).

We also repeated the denominator-free analysis based only on synchronous response rates for the obviously asynchronous SOAs. After excluding participants with pooled SRR ratio > 1, the pooled obvious-SOA-only SRR effect was attenuated ($U = 337.0$, $p = 0.081$, rank-biserial $r = 0.284$, 95% CI [-0.030, 0.581]). The AF obvious-SOA-only effect remained nominally significant but did not survive FDR

correction across the two condition-specific comparisons (*U* = 344.5, *p* = 0.027, *q*FDR = 0.055, rank-biserial *r* = 0.312, 95% CI [0.046, 0.573]), whereas the VF effect was non-significant (*U* = 311.5, *p* = 0.231, *q*FDR = 0.231, rank-biserial *r* = 0.187, 95% CI [-0.124, 0.478]).

We propagated the SRR ratio > 1 sensitivity analysis to the behavior-beta PLS model. In the full behavior-beta sample, LV1 was significant by permutation testing (N = 46, permutation p = 0.006), with a positive association between brain and behavioral latent scores (r = 0.421, p = 0.0036). After excluding participants with pooled SRR ratio > 1, the latent-score association remained positive (N = 44, r = 0.355, p = 0.018), but the model-level permutation test no longer reached significance (permutation p = 0.192). Thus, the direction of the latent association was preserved, but evidence for the multivariate LV was attenuated.

Together, these sensitivity analyses indicate that the overall pooled SRR-ratio effect is partly sensitive to exclusion of participants with SRR ratios greater than 1. However, because SRR ratio > 1 captures the extreme but theoretically and empirically meaningful case (see *Results: Patients showed altered audiovisual simultaneity judgment*), we retained the full-sample SRR analysis as the primary test of over-binding at long SOAs. The AF-specific behavioral effect remained the most consistent result across ratio-based and denominator-free analyses. We therefore interpret SRR as audiovisual simultaneity-judgment behavior, particularly a tendency to report synchrony for highly asynchronous AF trials.

1.3. Descriptive characterization of participants with SRR ratios greater than 1

To examine whether SRR ratios greater than 1 were more consistent with globally indiscriminate responding or potentially meaningful extreme behavior, we characterized the three patients who showed SRR > 1 in at least one condition (MEG030, MEG034, and MEG040). Only MEG034 showed SRR > 1 in both AF and VF conditions, whereas MEG030 and MEG040 exceeded 1 in only one condition. To further characterize these participants, we examined whether these were isolated behavioral anomalies or they also occupied high values on independently derived speech and clinical dimensions. Descriptive investigation showed that these participants occupied the high end of the behavior-beta LV1 beta-score distribution and the SRR LV1 score distribution. Rank-biserial comparisons between participants with any condition-specific SRR ratio > 1 and participants with SRR ratio <= 1 showed large positive effects for behavior-beta LV1 beta score (rank-biserial r = 0.91, 95% CI [0.69, 1.00]), SRR LV1 score (rank-biserial r = 1.00, 95% CI [1.00, 1.00]), semantic PC (rank-biserial r = 0.86, 95% CI [0.63, 1.00]), and syntactic PC (rank-biserial r = 0.94, 95% CI [0.81, 1.00]).

Within the patient sample, these 3 SRR > 1 participants also tended to show higher clinical PC1 scores than SRR <= 1 patients (SRR > 1 median = 4.77; SRR <= 1 median = 0.73; rank-biserial r = 0.67, 95% CI [0.15, 1.00], Mann-Whitney p = 0.079), although this clinical comparison was descriptive and did not reach conventional statistical significance. Because speech and clinical PCs were derived outside the MEG simultaneity-judgment task, these patterns argue against interpreting SRR ratio > 1 values solely as task disengagement, random responding, or response-mapping error. At the same time, because behavior-beta associations were attenuated after excluding pooled SRR ratio > 1 participants, SRR-dependent findings are retained as full-sample results and require replication in larger samples.

2. Psychophysical behavioral analysis/results

2.1. Methods

The pooling of trials into conditions yielded a total of 22 conditions with an associated response rate values per participant: 11 SOA and 2 categories of trial sequencing (AF and VF). The SRR values for each category are used to derive psychometric curves by fitting the mean, standard deviation, and amplitude of a Gaussian model to the response rate values (in %). The Gaussian model's mean parameter corresponds to the point of subjective simultaneity for AF (Point of Subjective Simultaneity (PSS) A) and VF (PSS V) conditions. Temporal recalibration (TR), estimated as the difference between PSS A and PSS V values, reflects rapid adjustments in perceptual processing made to align asynchronous audio-visual sensory inputs together and facilitate multi-modal sensory integration (*97*).

We derived the parameters of Gaussian models for each subject in both AF and VF conditions alongside their associated Gaussian curves using all SOA values and repeated the process with only ambiguous conditions. In all cases, participants who estimated TR magnitude exceeded the realistic range (±300ms) or for whom the curve fitting algorithm did not converge for either AF or VF conditions were excluded from analyses involving the associated PSS and TR values. For visualization purposes, we also derived group-average PSS and TR values by averaging the SSR of all participants that were not rejected in each group and fitting a Gaussian model to the resulting SSR values. We tested the significance of TR values in each group (one-sample *t*-test against 0).

2.2. Results

When using all SOA conditions, no control was excluded, and 3 patients were excluded due to non-converging curve fitting or TR values exceeding the realistic range. The TR values for HC were significant (one-sample *t* test against 0, $t(24)=4.237$, $p=0.0003$) with an average of 30ms, replicating results from previous studies (Figure S1, top right). The TR values for the patient group were also significant (one-sample *t* test against 0, $t(22)=2.484$, $p=0.0231$), with an average of 41ms (Figure S1, bottom right). No significant differences were found between groups for any of the PSS or TR measures using two-sample independent *t* tests (Welch's two-sample t-test, PSS_VF: $t(44.89) = 1.55$, $p = 0.13$; PSS_AF: $t(42.90) = 0.95$, $p = 0.35$; TR: $t(30.62) = 1.16$, $p = 0.26$).

When using only ambiguous SOA conditions, 1 HC participant was excluded and 4 patients were excluded due to non-converging curve fitting or TR values exceeding realistic range. The TR values for HC remained significant (one-sample *t* test against 0, $t(23)=4.124$, $p=0.0004$) with a slightly lower average of 22 ms. The TR values for the patient group were no longer significant (one-sample *t* test against 0, $t(21)=1.20$, $p=0.2365$), with an average of 22 ms. No significant differences were found between groups for any of the PSS or TR measures using two-sample independent *t* tests.

3. Cluster-based permutation test on ERSP maps between patients and controls

3.1. Method

To assess group differences in event-related spectral perturbations (ERSPs) without imposing a priori constraints on time-frequency windows, we performed non-parametric cluster-based permutation tests using FieldTrip. Source-localized ERSP maps were extracted from individualized peak vertices within bilateral primary motor cortex (M1), inferior frontal gyrus (IFG), primary auditory cortex (A1), and primary visual cortex (V1) for motor, auditory, and visual events, respectively. ERSPs were computed across frequencies spanning 15-30 Hz and time windows from -0.5 to 1 s relative to event onset. Prior to statistical testing, the time dimension was down-sampled to ~20-ms bins to improve computational efficiency while preserving temporal resolution. Group differences between healthy controls (HC; $n = 25$) and patients with schizophrenia (SCZ; $n = 23$) were tested using independent-samples t-statistics with cluster-based correction for multiple comparisons across time and frequency. Clusters were formed by identifying adjacent time-frequency samples exceeding a cluster-forming threshold of $p < 0.05$ (two-sided). Cluster-level statistics were computed using the maximum cluster mass (sum of t-values), and significance was assessed via Monte Carlo permutation with 1,000 randomizations. The family-wise error rate was controlled at $\alpha = 0.05$ (two-tailed). This approach provides robust control over multiple comparisons while remaining sensitive to spatially and temporally extended effects.

3.2. Result

Cluster-based permutation testing on time-frequency representations (15-30 Hz, -0.5 to 1 s) identified significant group differences in beta-band activity for both motor- and sensory-locked events (Figure S2). For motor events, significant clusters were observed in primary motor cortex contralateral to the moving hand (RM1 for left-hand movements, LM1 for right-hand movements) and in inferior frontal gyrus (LIFG and RIFG). These clusters were temporally extended over the peri- and post-movement interval (≈0~1 s) and indicated delayed and attenuated post-movement beta rebound in patients relative to controls. For sensory events, significant beta clusters were found in right primary auditory cortex (RA1) following

auditory onsets (≈0~0.5 s) and in right primary visual cortex (RV1) from pre-visual to around visual onsets (-0.5~0.2s). These sensory clusters reflected weaker event-related beta suppression in patients. Although cluster-based tests are conservative and favor temporally and spectrally extended effects (*98–100*), the presence of significant clusters in both motor and sensory cortices indicates that the observed beta abnormalities are robust at the group level, with motor-related differences showing the broadest temporal extent.

3.3. Comparison of predefined sensory windows and cluster-based sensory beta results

The predefined sensory beta windows were selected to test early post-stimulus beta modulation in visual and auditory cortices. The trending group difference in visual beta suppression was observed within the predefined early visual window (0-0.2 s). In contrast, the predefined early auditory window did not show a significant group difference. A group difference in auditory beta suppression was instead identified using cluster-based permutation testing across the beta-band time-frequency map, emerging at a later latency of approximately 0.2-0.5 s after auditory onset. This indicates that the auditory beta effect should be interpreted as a cluster-based, later-latency effect rather than as confirmation of the original early auditory-window hypothesis.

This later timing is nevertheless physiologically plausible. Early auditory responses around ~100 ms are commonly associated with obligatory stimulus-driven encoding, whereas auditory perception and audiovisual temporal judgments require integration of information over longer temporal windows, including extraction of temporal structure, comparison with predictions, and assessment of cross-modal coincidence (*98*, *99*, *101*). Prior work also suggests that expectation- and prediction-related auditory responses can unfold over later post-stimulus intervals rather than being limited to the earliest auditory response (*102*, *103*). Accordingly, the 0.2-0.5 s auditory beta cluster may reflect altered sustained auditory-locked or post-sensory predictive evaluation in schizophrenia, rather than a robust deficit in initial auditory encoding. This auditory result should therefore be interpreted cautiously and replicated in future work.

4. Baseline-control analysis for sensory-locked beta effects

Because the sensory baseline window occurred 1.5-1.0 s before the current sensory event, it could overlap with residual post-movement activity from the previous trial. We therefore tested whether group differences in absolute beta power during the sensory baseline window could account for group differences in post-sensory beta modulation.

For each sensory-locked analysis, we compared absolute beta power between groups during the baseline period used for ERSP normalization. No significant group differences were observed for baseline beta power in left V1 or bilateral IFG for visual-locked events (LV1: $t(30.35) = -1.80$, $p = 0.08$; LIFG: $t(45.54) = -1.27$, $p = 0.21$; RIFG: $t(45.66) = -0.97$, $p = 0.34$), or in bilateral A1 and bilateral IFG for auditory-locked events (LA1: $t(45.97) = -1.26$, $p = 0.22$; RA1: $t(41.95) = -0.94$, $p = 0.35$; LIFG: $t(45.64) = -0.34$, $p = 0.74$; RIFG: $t(45.80) = -0.05$, $p = 0.96$).

The only trending baseline difference was observed in right V1 during visual-locked events, where patients showed *higher* absolute baseline beta power than controls (RV1: $t(41.61) = -2.21$, $p = 0.03$, assuming group coding of controls minus patients). This direction is inconsistent with the possibility that reduced sensory beta suppression in patients resulted from lower baseline beta power. Overall, these baseline-control analyses indicate that the sensory beta findings are unlikely to be explained by group differences in absolute beta power during the sensory baseline period.

5. Leave-one-participant-out robustness of reported PLS models

We assessed whether the three reported PLS models were driven by individual high-leverage observations using leave-one-participant-out (LOO) sensitivity analyses. For the audiovisual simultaneity behavior-beta model, the full-sample LV1 latent-score correlation was $r = 0.421$, permutation $p = 0.006$. Across LOO

iterations, the Pearson LV1 correlation remained positive in 46/46 iterations, with mean r = 0.422, SD = 0.014, and range = [0.362, 0.448]. The beta-feature weight pattern remained highly similar to the full-sample solution across LOO iterations, with mean pattern correlation $r = 0.997$, $SD = 0.006$, and range = [0.974, 1.000]. No participant was flagged as potentially influential.

For the semantic PC-beta model, the full-sample LV1 latent-score correlation was $r = 0.501$, permutation $p = 0.036$. Across LOO iterations, the Pearson LV1 correlation remained positive in 48/48 iterations, with mean r = 0.503, SD = 0.008, and range = [0.473, 0.522]. The beta-feature weight pattern remained highly similar to the full-sample solution, with mean pattern correlation $r = 0.998$, $SD = 0.003$, and range = [0.988, 1.000]. No participant was flagged as potentially influential.

For the syntactic PC-beta model, the full-sample LV1 latent-score correlation was r = 0.468, permutation p = 0.044. Across LOO iterations, the Pearson LV1 correlation remained positive in 48/48 iterations, with mean $r = 0.471$, $SD = 0.011$, and range = [0.446, 0.515]. The beta-feature weight pattern remained highly similar to the full-sample solution, with mean pattern correlation $r = 0.997$, $SD = 0.007$, and range = [0.967, 1.000]. No participant was flagged as potentially influential. Together, these internal sensitivity analyses suggest that the reported PLS associations were not driven by any single participant, although they do not replace external validation in an independent cohort.

6. Group-residualized PLS sensitivity analysis

The primary PLS analyses were conducted across all participants to identify disease-relevant cross-domain covariance between event-related beta modulation and behavioral or language measures. Because diagnostic group differences may contribute to this covariance, we repeated the behavior-beta, semantic PC-beta, and syntactic PC-beta models after residualizing both beta features and behavioral/language variables for diagnostic group.

After group residualization, the behavior-beta model remained significant (LV1 permutation $p = 0.019$; latent-score $r = 0.388$), indicating that the association between audiovisual binding behavior and beta modulation was not explained solely by mean diagnostic group differences. In contrast, the semantic PC-beta model was no longer significant (LV1 permutation $p = 0.307$; latent-score $r = 0.324$), nor was the syntactic PC-beta model (LV1 permutation $p = 0.247$; latent-score $r = 0.356$). These results indicate that the primary language-beta findings reflect diagnosis-associated cross-domain covariance across the case-control sample, but do not provide evidence for diagnosis-independent individual-difference associations between beta modulation and semantic or syntactic speech organization.

7. Control-frequency analyses in the gamma band

To test whether the reported beta-band findings reflected a nonspecific event-related power abnormality across frequencies, we repeated the key analyses using event-related gamma-band power. We focused on gamma as the control frequency because gamma-band activity is commonly associated with feedforward sensory signalling and prediction-error processing, and gamma abnormalities are prominently implicated in schizophrenia (*39*, *62*). Gamma power was extracted from 31-60 Hz using the same task events, ROIs, and time windows as the beta-band features reported in the main text.

7.1. Gamma-band group differences

We first tested whether event-related gamma power showed patient-control differences across the same 32 event-related features used in the beta-band analyses. Welch's two-sample t-tests were conducted for each gamma feature. To match the multiple comparison correction logic used for the primary beta analyses, FDR correction was applied within analogous beta-outcome subfamilies: motor M1 pre-movement, motor M1 early post-movement, motor M1 rebound, motor IFG pre-movement, motor IFG early post-movement, motor IFG rebound, auditory A1 early, auditory IFG post-sensory, visual V1 early, and visual IFG post-

sensory. Motor subfamilies included 2 response hands × 2 hemispheres = 4 tests each, and sensory subfamilies included left and right hemispheric targets = 2 tests each.

Five gamma features showed nominal uncorrected group differences at $p < 0.05$. The strongest effect was observed for left M1 gamma rebound following left-hand movement, where patients showed lower gamma power than controls (PT mean = −0.52, HC mean = 4.43; Welch's $t(37.38) = -3.05$, $p = 0.0042$, qFDR = 0.017, Hedges' $g = -0.85$). This one effect survived FDR correction within the motor M1 rebound subfamily. Additional nominal effects were observed for motor.RIFG.Lhand.gamma.early ($t(38.67) = -2.31$, $p = 0.026$, qFDR = 0.076), audio.RA1s.gamma.early ($t(40.59) = -2.28$, $p = 0.028$, qFDR = 0.056), motor.RM1.Lhand.gamma.rebound ($t(29.38) = -2.17$, $p = 0.038$, qFDR = 0.076), and motor.LIFG.Lhand.gamma.early ($t(26.05) = -2.18$, $p = 0.038$, qFDR = 0.076), but these effects did not survive subfamily FDR correction.

The gamma-band control analysis identified a localized corrected group difference in M1 gamma rebound, indicating that movement-related rebound abnormalities were not exclusively beta-band-specific. However, the broader distributed beta-band pattern was not reproduced in gamma: corrected gamma effects were limited to one M1 rebound feature.

7.2. Gamma-band PLS analyses

We next repeated the three primary PLS analyses using gamma-band features instead of beta-band features. These analyses paralleled the models reported in the main text: audiovisual binding behavior, semantic PC, and syntactic PC. The gamma-band behavior model did not yield a significant latent variable (LV1: permutation $p = 0.1984$; LV2: permutation $p = 1.0000$). Similarly, the gamma-band semantic PC model was not significant (LV1: permutation $p = 0.2336$), and the gamma-band syntactic PC model was not significant (LV1: permutation $p = 0.8778$).

Thus, the key beta-band associations with audiovisual temporal binding behavior and theory-driven semantic and syntactic speech components were not reproduced in the gamma band. These control-frequency analyses suggest that the reported multivariate associations are unlikely to reflect a nonspecific relationship between event-related power and behavioral or language measures across frequency bands. However, because this analysis tested one control frequency band, the results should be interpreted as supporting the relative prominence of beta-band effects rather than proving absolute beta specificity.

8. Control-region analysis using bilateral S_orbital_med-olfact

To test whether the reported beta-band findings reflected spatially-specific event-related beta changes, we conducted a control-region analysis using bilateral S_orbital_med-olfact. This region was selected as a non-task-relevant cortical control region outside the a priori sensory-motor-IFG network hypothesized to support (audio/visual) perception-action-language integration.

8.1. Control ROI beta extraction

For each participant, source-level epoched MEG data were projected to the default anatomy surface in Brainstorm. Time-frequency power was computed using Morlet wavelets from 1 to 60 Hz for all vertices within left and right S_orbital_med-olfact. Event-related perturbation was computed using the same ERS/ERD normalization approach as in the main event-related analyses. Motor-locked control ROI features were baseline-normalized using the -2.5 to -2.0 s pre-movement window, and sensory-locked control ROI features were baseline-normalized using the -1.5 to -1.0 s pre-stimulus window. Within each hemisphere, beta power was averaged across all vertices within the control ROI, beta frequencies, and the corresponding event-related time windows.

Motor-locked control ROI features were extracted for left- and right-hand responses using the same windows as the primary motor analyses: pre-movement beta modulation (-0.5 to 0 s), early post-movement

beta modulation (0 to 0.5 s), and post-movement beta rebound (0.5 to 1.0 s). This yielded 12 motor control ROI beta features: two hemispheres × two response hands × three time windows. Sensory-locked control ROI features were extracted for auditory-locked and visual-locked events using the same sensory windows as the primary analyses: the early sensory window (0 to 0.2 s) and the later post-sensory window (0.2 to 0.6 s), separately for left and right control ROIs. This yielded 8 sensory control ROI beta features: two hemispheres × two sensory modalities × two time windows. In total, 20 control ROI beta features were extracted.

8.2. Control ROI event-related group comparisons

We first tested whether control ROI beta modulation reproduced the patient-control group differences observed in the theory-driven sensory-motor-IFG regions. Group comparisons were conducted for each of the 20 control ROI beta features using Welch's two-sample t-tests. FDR correction was applied within analogous beta-outcome subfamilies matching the primary event-related beta analysis: motor pre-movement, motor early post-movement, motor rebound, visual early, visual late/post-sensory, auditory early, and auditory late/post-sensory. Motor subfamilies included 2 response hands × 2 control-ROI hemispheres = 4 tests each, and sensory subfamilies included left and right control-ROI hemispheres = 2 tests each.

No patient-control group differences were observed in bilateral S_orbital_med-olfact beta modulation. None of the 20 control ROI beta features reached nominal significance or survived FDR correction (t = -1.66 to 1.64; p = 0.106 to 0.840). The smallest uncorrected effect was observed for left control ROI beta rebound following left-hand responses, but this effect was not significant after subfamily correction (PT mean = 0.86, HC mean = 5.30; Welch's $t(38.63) = -1.66$, $p = 0.106$, qFDR = 0.254, Hedges' $g = -0.46$). Thus, the event-related beta group differences observed in the theory-driven sensory-motor-IFG network were not reproduced in the control ROI analysis.

9. Exploratory correlations between PANSS-G5 and motor beta features

Given that PANSS-G5 contributed to the clinical illness-burden component, we conducted an exploratory follow-up analysis to examine whether PANSS-G5 was strongly associated with any individual motor beta feature. Pearson correlations were computed between PANSS-G5 and all beta features containing "motor" in the feature name. This analysis was intended only as a descriptive check and was not corrected for multiple comparisons.

Across motor beta features, correlations with PANSS-G5 were small to modest in magnitude, ranging from $r = -.28$ to $r = 0.26$. Uncorrected p-values ranged from $p = 0.0509$ to $p = 0.993$. Thus, no individual motor beta feature showed strong evidence of a robust association with PANSS-G5. The observed clinical association with the beta latent score should not be interpreted as being specifically driven by PANSS-rated motor mannerisms/posturing, and catatonia and psychomotor symptom-specific scales (*104*) are required to unpack this hypothesis.

10. Exploratory IFG-to-sensory/motor beta-band PTE analysis

10.1. Method

To explore whether sensory and motor beta modulation was related to IFG-linked directed network dynamics, we conducted an exploratory beta-band phase-transfer entropy (PTE) analysis (*105*). This analysis was not used as primary evidence for the main conclusions. PTE was estimated over a broad 0–1 s post-event activation window to provide sufficient temporal support for beta-band entropy estimation. Because this window is broader than several beta-power outcome windows, particularly early sensory beta suppression, the results should not be interpreted as temporally specific evidence that IFG activity drives transient beta modulation within those shorter windows.

Activation-minus-baseline PTE was extracted for ipsilateral IFG→target and target→IFG connections. Target regions were M1 for movement-locked beta modulation, A1 for auditory-locked beta modulation, and V1 for visual-locked beta modulation. IFG subregions were averaged within hemisphere before extracting directed connectivity values. Across all participants, standardized group-adjusted linear models tested whether IFG→target PTE predicted beta modulation in the corresponding target cortex.

The 12 target-aligned regression tests were organized according to the same beta-outcome subfamilies used for the event-related beta analyses: M1 early beta modulation outcomes (2 response hands × 2 M1 hemispheres = 4 tests), M1 post-movement beta rebound (PMBR) outcomes (2 response hands × 2 M1 hemispheres = 4 tests), auditory A1 beta modulation outcomes (left and right A1 hemispheric targets = 2 tests), and visual V1 beta modulation outcomes (left and right V1 hemispheric targets = 2 tests). FDR correction was applied within each beta-outcome subfamily, separately for each PTE model family: forward IFG→target models, reverse target→IFG models, directionality-index models, patient-only forward models, and group-by-PTE interaction models.

10.2. Forward IFG→target models

Forward IFG→target models showed significant associations in 8 of 12 target-aligned tests after FDR correction within beta-outcome subfamilies. Significant associations were observed for movement-locked M1 beta modulation, bilateral V1 visual beta modulation, and left A1 auditory beta modulation.

For movement-locked outcomes, significant associations included right-hand RM1 early beta modulation (RIFG→RM1: standardized $\beta = -0.63$, $F(1,45) = 29.20$, $p < 0.001$, qFDR < 0.001, partial $R^2 = 0.394$), right-hand RM1 PMBR (RIFG→RM1: $\beta = -0.52$, $F(1,45) = 16.58$, $p < 0.001$, qFDR < 0.001, partial $R^2 = 0.269$), right-hand LM1 early beta modulation (LIFG→LM1: $\beta = -0.47$, $F(1,45) = 13.14$, $p < 0.001$, qFDR = 0.001, partial $R^2 = 0.226$), left-hand LM1 early beta modulation (LIFG→LM1: $\beta = -0.42$, $F(1,45) = 10.27$, $p = 0.002$, qFDR = 0.003, partial $R^2 = 0.186$), and left-hand RM1 early beta modulation (RIFG→RM1: $\beta = -0.42$, $F(1,45) = 9.84$, $p = 0.003$, qFDR = 0.003, partial $R^2 = 0.179$).

For sensory-locked outcomes, significant associations were observed for visual beta modulation in bilateral V1 (LIFG→LV1: $\beta = -0.44$, $F(1,45) = 9.25$, $p = 0.004$, qFDR = 0.008, partial $R^2 = 0.171$; RIFG→RV1: $\beta = -0.39$, $F(1,45) = 7.45$, $p = 0.009$, qFDR = 0.009, partial $R^2 = 0.142$) and for left auditory beta modulation (LIFG→LA1: $\beta = -0.34$, $F(1,45) = 6.03$, $p = 0.018$, qFDR = 0.036, partial $R^2 = 0.118$). Full statistics for all forward IFG→target models are reported in Table S3.

10.3. Reverse-direction and directionality-index models

As a directionality control, we tested reverse target→IFG PTE models. None of the 12 reverse-direction models survived FDR correction, and none reached nominal significance. Full reverse-direction statistics are reported in Table S4.

We also computed a directionality index, defined as IFG→target PTE minus target→IFG PTE. Directionality-index models showed significant associations in 7 of 12 tests after FDR correction within beta-outcome subfamilies. These included RM1 right-hand early beta modulation ($\beta = -0.58$, $F(1,45) = 22.31$, $p < 0.001$, qFDR < 0.001, partial $R^2 = 0.331$), bilateral V1 visual beta modulation, left A1 auditory beta modulation, and several M1 early beta modulation features. Full directionality-index statistics are reported in Table S5.

10.4. Patient-only and group-by-PTE interaction models

Patient-only forward models showed a similar but more limited pattern. Four associations survived FDR correction within beta-outcome subfamilies: RIFG→RM1 PTE predicted RM1 right-hand early beta modulation ($\beta = -0.70$, $F(1,21) = 20.07$, $p < 0.001$, qFDR < 0.001, partial $R^2 = 0.489$); LIFG→LV1 PTE predicted LV1 visual beta modulation ($\beta = -0.64$, $F(1,21) = 14.57$, $p = 0.001$, qFDR = 0.002, partial $R^2 =$

0.410); LIFG→LM1 PTE predicted LM1 right-hand early beta modulation (β = -0.50, $F(1,21) = 6.96$, p = 0.015, qFDR = 0.031, partial $R^2 = 0.249$); and RIFG→RM1 PTE predicted RM1 left-hand early beta modulation (β = -0.46, $F(1,21) = 5.59$, $p = 0.028$, qFDR = 0.037, partial $R^2 = 0.210$). Additional motor rebound associations were nominal but did not survive FDR correction in the patient-only sample.

Group-by-PTE interaction models were generally non-significant after FDR correction, except for the LIFG→LV1 visual beta model (interaction $\beta = -0.89$, $F(1,44) = 11.16$, $p = 0.0017$, qFDR = 0.0034, partial $R^2 = 0.202$). This suggests that most IFG→target PTE–beta associations were not explained solely by group-specific slopes, although these interaction analyses remain exploratory.

10.5. Interpretation

Together, these exploratory analyses indicate that beta modulation in sensory and motor target cortices was more consistently associated with IFG→target beta-band PTE than with reverse target→IFG PTE. However, these findings should be interpreted cautiously. PTE reflects directed statistical dependence in phase dynamics rather than causal perturbation evidence. In addition, because PTE was estimated over a broad 0–1 s post-event window, it was not temporally matched to all target beta-power outcomes, including early sensory beta suppression windows. Therefore, these results do not establish temporally precise top-down control of transient beta modulation. Instead, they provide exploratory evidence that IFG-linked directed statistical dependence covaries with sensory and motor beta modulation.

Although PTE and beta-power modulation were derived from beta-band source activity in overlapping event-related windows, they quantify distinct signal properties: PTE indexes directed phase-based statistical dependence between IFG and target regions, whereas beta modulation indexes within-region power change relative to baseline.

11. Exploratory clinical correlations with positive and negative symptom dimensions

Recent meta-analytic evidence suggests that direct associations between audiovisual multisensory integration measures and clinical symptoms in schizophrenia are typically small, despite group-level MSI abnormalities (*78*). We therefore conducted exploratory patient-only analyses to contextualize the relationships among clinical severity, multisensory behavior, speech organization, and beta modulation.

To address symptom specificity, positive- and negative-symptom components were estimated separately. The positive-symptom component was derived from PANSS P1, P2, P3, and G9, and explained 63.2% of variance across these items. Loadings were P1 = 0.59, P2 = 0.21, P3 = 0.49, and G9 = 0.61. The negative-symptom component was derived from PANSS N1, N4, and N6, and explained 47.3% of variance. Loadings were N1 = 0.72, N4 = 0.65, and N6 = 0.25. Component signs were oriented so that higher scores reflected greater symptom severity.

These symptom components, together with the overall illness-burden component reported in the main text, were correlated with the major behavioral, speech, and neural summary metrics: SRR_A, SRR_V, pooled SRR, semantic PC, syntactic PC, semantic-beta latent score, and syntactic-beta latent score. Pearson correlations were computed as the primary exploratory analysis, and false-discovery-rate correction was applied across the correlation matrix. Because SRR measures were non-normally distributed, Spearman correlations were also examined as sensitivity analyses. See Table S7 for the full correlation matrix.

No exploratory correlation survived FDR correction in either the Pearson or Spearman analyses. Direct correlations between SRR measures and clinical severity were small and non-significant. Across illness-burden, positive-symptom, and negative-symptom components, correlations with SRR_A, SRR_V, and pooled SRR were all small in magnitude (Pearson $|r| \leq 0.18$, all $p \geq 0.43$), consistent with prior meta-analytic evidence that direct MSI-clinical associations in schizophrenia are typically weak.

For speech and beta-summary metrics, the strongest nominal associations involved the positive-symptom component. Positive symptoms were associated with syntactic PC scores (Pearson $r = 0.50$, $p = 0.014$, qFDR = 0.308; Spearman ρ = 0.49, $p = 0.018$, qFDR = 0.281) and syntactic-beta latent scores (Pearson $r = 0.45$, $p = 0.029$, qFDR = 0.308; Spearman ρ = 0.45, $p = 0.031$, qFDR = 0.281), although these associations did not survive FDR correction. Positive symptoms also showed a trend-level association with semantic-beta latent scores (Pearson $r = -0.41$, $p = 0.053$, qFDR = 0.308; Spearman ρ = -0.40, $p = 0.060$, qFDR = 0.281). Negative-symptom associations with semantic-beta and syntactic-beta latent scores were weaker and non-significant (semantic-beta: Pearson $r = -0.31$, $p = 0.150$, qFDR = 0.450; syntactic-beta: Pearson $r = 0.29$, $p = 0.177$, qFDR = 0.465).

Overall, these exploratory analyses do not support a robust direct association between behavioral MSI measures and clinical severity in this sample. Instead, the strongest nominal symptom-specific patterns involved syntactic organization and syntactic-beta latent scores in relation to positive/disorganization-spectrum symptoms. Given the modest patient-only sample size and the absence of FDR-significant effects, these analyses should be interpreted cautiously and treated as descriptive support for future studies rather than as evidence for symptom-specific biomarkers.

12. Medication-sensitivity analyses for beta modulation

Although antipsychotic exposure did not correlate with the illness-burden component in the main analysis, this does not rule out a direct relationship between medication exposure and beta modulation. We therefore conducted patient-only medication-sensitivity regressions to test whether antipsychotic exposure predicted beta measures after controlling for illness severity. Antipsychotic exposure was quantified as Defined Daily Dose (DDD), the assumed average maintenance dose per day for a drug used for its main indication in adults. For each model, the beta outcome was regressed on antipsychotic DDD and the illness-burden PC:

beta outcome ~ antipsychotic DDD + illness-burden PC

The medication term was the effect of interest. We tested three levels of beta outcome: PC1 extracted from motor-related beta features, PC1 extracted from sensory-related beta features, individual event-related beta features, and the beta latent scores from the behavior-beta, semantic-beta, and syntactic-beta PLS models. PC1 was extracted separately from motor-related and sensory-related beta features to provide dimensionality-reduced measures of beta modulation in each task domain. The motor-related beta PC1 was derived from 24 movement-related beta features and explained 37.1% of their variance; the sensory-related beta PC1 was derived from 8 sensory-locked beta features and explained 48.0% of their variance.

Antipsychotic DDD did not predict the motor-related beta PC1 after controlling for illness burden (standardized $\beta = -0.009$, $F(1,20) = 0.002$, $p = 0.968$, partial $R^2 < 0.001$), nor did it predict the sensory-related beta PC1 (standardized $\beta = 0.165$, $F(1,20) = 0.582$, $p = 0.454$, partial $R^2 = 0.028$). At the individual-feature level, no medication effect survived FDR correction across motor or sensory beta features. The strongest uncorrected motor-feature association was observed for RIFG left-hand beta rebound (standardized $\beta = -0.418$, $F(1,20) = 4.238$, $p = 0.053$, partial $R^2 = 0.175$, qFDR = 0.998). The strongest uncorrected sensory-feature association was observed for right A1 early auditory beta modulation (standardized $\beta = 0.272$, $F(1,20) = 1.603$, $p = 0.220$, partial $R^2 = 0.074$, qFDR = 0.871).

Finally, antipsychotic DDD did not predict the PLS beta latent scores after controlling for illness burden. This was true for the behavior-beta latent score (standardized β = 0.117, F(1,20) = 0.291, p = 0.596, partial $R^2 = 0.014$), semantic-beta latent score (standardized $\beta = 0.077$, $F(1,20) = 0.131$, $p = 0.721$, partial $R^2 = 0.007$), and syntactic-beta latent score (standardized $\beta = -0.083$, $F(1,20) = 0.154$, $p = 0.699$, partial $R^2 = 0.008$). Together, these analyses did not provide evidence that antipsychotic exposure, quantified as DDD, explained the reported beta modulation findings. However, given the modest patient-only sample size and observational design, medication effects cannot be fully ruled out.

Supplementary Figures

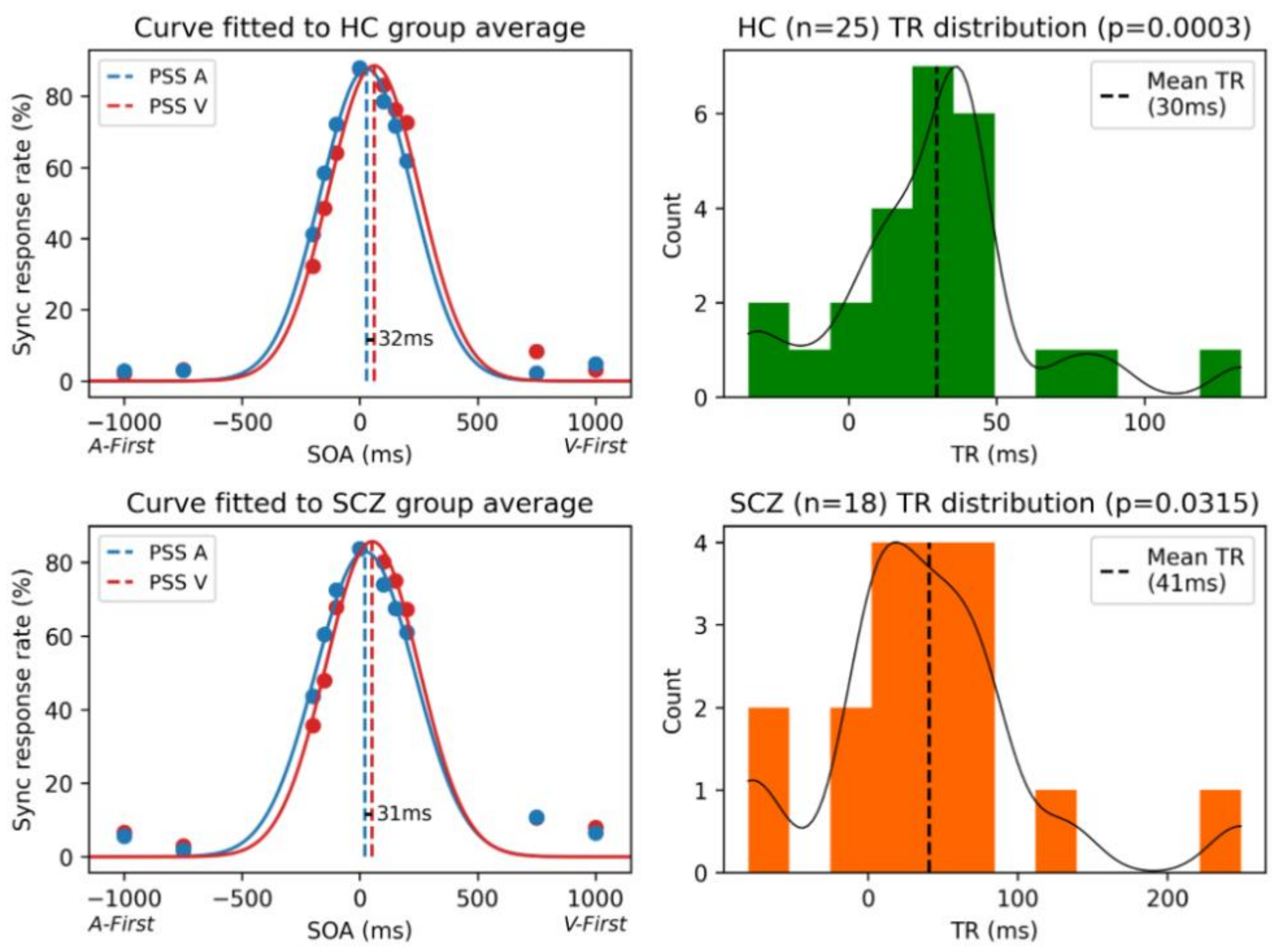

Figure S1. Summary of temporal recalibration analysis using all SOA conditions for both groups. The left column illustrates the gaussian models (solid lines) fitted to HC (top) and SCZ (bottom) group-averaged SSR values, with their respective PSS (dashed lines) alongside the associated temporal recalibration (black line). The right column illustrates the distribution of temporal recalibration values (histogram) for HC (top) and SCZ (bottom) alongside a matching kernel density estimate (solid black line) and the average TR (dashed line).

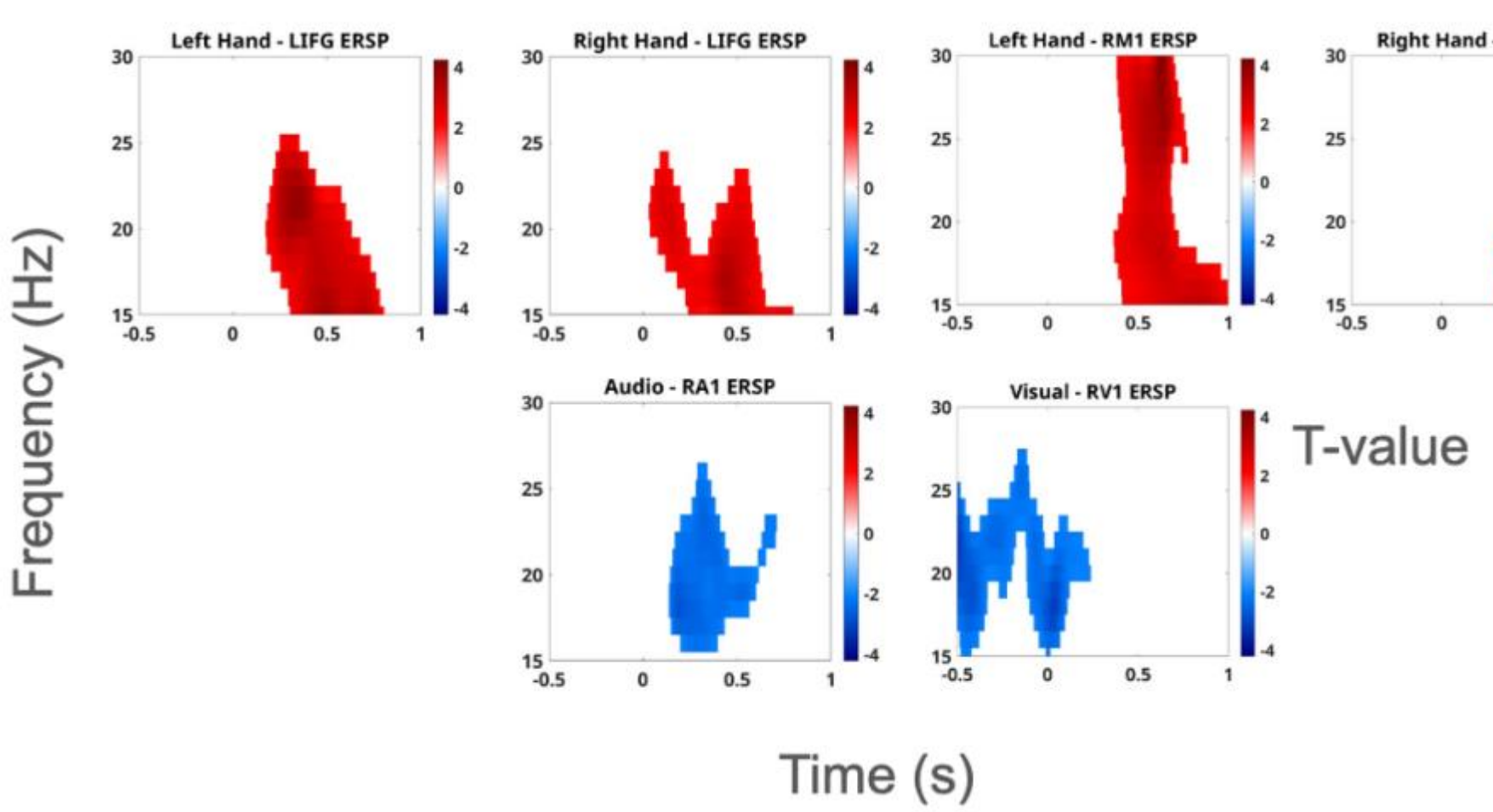

Figure S2. Cluster-based permutation results for beta-band activity.
Significant time-frequency clusters (15-30 Hz) identified by cluster-based permutation testing comparing patients with schizophrenia and healthy controls are shown for motor-locked (left and right hand movements) and sensory-locked (auditory and visual onsets) events. Color values represent t-statistics for group differences, with red indicating higher beta power in controls than patients and blue indicating lower beta power in controls than patients. For motor events, significant clusters were observed in primary motor

cortex (RM1 and LM1) and inferior frontal gyrus (LIFG and RIFG), spanning peri- and post-movement intervals and reflecting delayed and attenuated post-movement beta rebound in patients. For sensory events, significant clusters were observed in right primary auditory cortex (RA1) and right primary visual cortex (RV1), primarily during early post-stimulus intervals, reflecting weaker event-related beta suppression in patients. Axes denote time relative to event onset (s) and frequency (Hz).

Supplementary Tables

Table S1. Language PC loadings and speech feature definitions

Speech Domain	Feature Name	Loading on PC	Feature Description
**Semantics**	Consecutive Similarity	**.59**	Sentence-level cosine similarity. Higher similarity score indicates greater similarity between neighboring sentences in the embedding space.
	Perplexity	**-.62**	Perplexity of a generative model when evaluating the text from a participant. Higher perplexity reflects a lower capacity of the model to predict the subject's word choice.
	Pseudo Perplexity	.23	Pseudo-perplexity represents the ability of a mask filling model to guess a content token*. Higher score is more unusual word choice given the preceding and following context
	Semantic Density	**-.46**	Ratio of meaningful to content words in a sentence, where meaningful components are derived via vector unpacking, applying gradient descent to word2vec sentence embeddings to approximate the original semantic vector. A higher value shows higher semantic density.
**Syntax**	Sentence Length	**-.55**	Number of words in a sentence.
	Syntax Depth	**-.54**	Depth of the syntax tree obtained by parsing a sentence through part-of-speech tagging. A higher value indicates a complex syntax.
	Clause Count	**-.63**	Number of clauses as recognized by pattern matching on the part-of-speech tags.

Note. Feature absolute loadings above 0.4 are bolded. * Pseudo-perplexity was computed using a multilingual masked language model by iteratively masking selected tokens and estimating the likelihood of the original token from the surrounding sentence context. In the implementation, selected tokens were intended to correspond to POS-defined meaningful words, including nouns, verbs, adjectives, adverbs, pronouns, numbers, and proper nouns. Token selection was implemented by excluding token IDs associated with non-content words rather than by explicitly aligning subword tokens to POS-tagged source words. We therefore interpret this feature as an approximate POS-filtered masked-token predictability measure, with higher values reflecting lower contextual predictability among the tokens selected by the algorithm.

Table S2. NLP/LLM models for speech feature extraction

Feature	Feature domain	Model / parser used	Implementation details	English/French handling
**Consecutive sentence similarity**	Semantic coherence	intfloat/multilingual-e5-base	Sentences were tokenized with the corresponding HuggingFace tokenizer. Sentence-level embeddings were extracted from the first-token representation of the final hidden layer. Consecutive similarity was computed as the cosine similarity between adjacent sentence embeddings and averaged across the transcript.	This embedding model is multilingual and was applied to both English and French transcripts.
**Perplexity**	Lexical / sequential predictability	ibm-granite/granite-3.3-2b-base	The full transcript was tokenized with the corresponding HuggingFace tokenizer and passed to a decoder-only causal language model. Perplexity was computed as the exponentiated cross-entropy loss over next-token prediction.	The same decoder-only model was applied to English and French transcripts. Because the model was not used as a language-specific normative benchmark, perplexity values were interpreted comparatively within the study rather than as absolute language-specific estimates.
**Semantic density**	Semantic richness / compactness	Vocabulary-restricted version* of the pre-trained Google News Word2Vec model (word2vec-google-news-300), implemented using Gensim	Content words were selected using POS tags, lemmatized, lowercased, and retained only if present in the Word2Vec vocabulary. Sentence-level semantic vectors were calculated by averaging retained word vectors. A gradient-based reconstruction procedure estimated the number of meaning-bearing components required to approximate each sentence vector; semantic density was calculated as the number of selected meaning components divided by the number of retained content words.	The same Gensim Word2Vec model was applied to English and French transcripts. Because Gensim is the implementation rather than a language-specific model family, bilingual coverage depends on the vocabulary of the specific Word2Vec model; therefore, only lemmatized content words present in the model vocabulary were retained.
**Sentence length**	Syntactic production quantity	stanza	Sentences were parsed using stanza.Pipeline(lang, processors="tokenize,mwt,pos,lemma"). Sentence length was calculated as the number of parsed tokens per sentence and averaged across the transcript.	Each subject consistently used English or French in the speech task. Transcript language was assigned at the file level: files containing "FR" were processed as French (lang="fr"), and all other files were processed as English (lang="en"). English analyses used Penn-style xpos tags; French analyses used Universal POS upos tags.
**Syntax depth**	Syntactic complexity	stanza + nltk.RegexpParser	POS-tagged and lemmatized tokens were passed to nltk.RegexpParser using language-specific grammar rules. Syntax depth was calculated as the height of each resulting chunk	Separate English and French grammar rules were used. English parsing used Penn-style xpos tags; French parsing used Universal POS upos tags.

			tree and averaged across sentences.	
**Clause count**	Syntactic complexity	stanza + nltk.RegexpParser	Clause count was calculated as the number of chunk-tree subtrees labelled CLAUSE in each sentence and averaged across sentences.	Separate English and French chunking rules were used. English parsing used Penn-style xpos tags; French parsing used Universal POS upos tags.

*Word embeddings were derived from a vocabulary-restricted version of the pre-trained Google News Word2Vec model (word2vec-google-news-300), implemented using Gensim. The original model contains 300-dimensional vectors trained on the Google News corpus. To reduce the vocabulary for semantic density estimation, the original model was filtered to retain only common English and French words. Specifically, lists of the 50,000 most frequent English and French words were downloaded from the hermitdave/FrequencyWords repository; the top 15,000 words from each language were lemmatized using Stanza, lowercased, deduplicated, and used as a candidate vocabulary. Only candidate words present in the original Google News Word2Vec model were retained. The resulting reduced model was saved as a Gensim KeyedVectors object and contained 300-dimensional vectors for 15,855 vocabulary items. This vocabulary restriction was applied to improve semantic density estimation by avoiding optimization over the full Google News vocabulary of approximately 3 million tokens, which included many irrelevant or noisy entries. The reduced model was saved using Gensim 4.3.3 and Python 3.12.9.

Table S3. Forward IFG→target PTE models predicting target beta modulation.
Standardized group-adjusted linear models tested whether ipsilateral IFG→target activation-minus-baseline beta-band PTE predicted beta modulation in the corresponding sensory or motor target cortex. FDR correction was applied across the 12 target-aligned tests.

Domain	Target beta feature	PTE predictor	β	$F(1,45)$	p	qFDR	partial R^2
Motor	motor.LM1.Lhand.beta.early	ButtonL_LIFG_to_LM1_ΔPTE	-0.42	10.27	0.002	0.003	0.186
Motor	motor.RM1.Lhand.beta.early	ButtonL_RIFG_to_RM1_ΔPTE	-0.42	9.84	0.003	0.003	0.179
Motor	motor.LM1.Lhand.beta.rebound	ButtonL_LIFG_to_LM1_ΔPTE	-0.23	2.94	0.093	0.093	0.061
Motor	motor.RM1.Lhand.beta.rebound	ButtonL_RIFG_to_RM1_ΔPTE	-0.27	4.55	0.038	0.077	0.092
Motor	motor.LM1.Rhand.beta.early	ButtonR_LIFG_to_LM1_ΔPTE	-0.47	13.14	<0.001	0.001	0.226
Motor	motor.RM1.Rhand.beta.early	ButtonR_RIFG_to_RM1_ΔPTE	-0.63	29.20	<0.001	<0.001	0.394
Motor	motor.LM1.Rhand.beta.rebound	ButtonR_LIFG_to_LM1_ΔPTE	-0.26	3.53	0.067	0.089	0.073
Motor	motor.RM1.Rhand.beta.rebound	ButtonR_RIFG_to_RM1_ΔPTE	-0.52	16.58	<0.001	<0.001	0.269
Auditory	audio.LA1s.beta.early	sound_LIFG_to_LA1_ΔPTE	-0.34	6.03	0.018	0.036	0.118
Auditory	audio.RA1s.beta.early	sound_RIFG_to_RA1_ΔPTE	-0.11	0.55	0.463	0.463	0.012
Visual	visual.LV1s.beta.early	video_LIFG_to_LV1_ΔPTE	-0.44	9.25	0.004	0.008	0.171
Visual	visual.RV1s.beta.early	video_RIFG_to_RV1_ΔPTE	-0.39	7.45	0.009	0.009	0.142

Note. Negative β values indicate that stronger IFG→target PTE was associated with lower beta modulation in the corresponding target cortex. Eight of 12 forward IFG→target models survived FDR correction.

Table S4. Reverse target→IFG PTE models predicting target beta modulation.
Reverse-direction models tested whether target→IFG activation-minus-baseline beta-band PTE predicted beta modulation in the corresponding sensory or motor target cortex. FDR correction was applied across the 12 target-aligned tests. None of the reverse target→IFG models reached nominal significance or survived FDR correction.

Domain	Target beta feature	PTE predictor	β	F(1,45)	p	qFDR	partial R^2
Motor	motor.LM1.Lhand.beta.early	ButtonL_LM1_to_LIFG_ΔPTE	-0.05	0.14	0.709	0.945	0.003
Motor	motor.RM1.Lhand.beta.early	ButtonL_RM1_to_RIFG_ΔPTE	-0.13	0.77	0.384	0.768	0.017
Motor	motor.LM1.Lhand.beta.rebound	ButtonL_LM1_to_LIFG_ΔPTE	-0.11	0.60	0.443	0.443	0.013
Motor	motor.RM1.Lhand.beta.rebound	ButtonL_RM1_to_RIFG_ΔPTE	-0.17	1.54	0.221	0.316	0.033
Motor	motor.LM1.Rhand.beta.early	ButtonR_LM1_to_LIFG_ΔPTE	-0.20	1.87	0.179	0.716	0.040
Motor	motor.RM1.Rhand.beta.early	ButtonR_RM1_to_RIFG_ΔPTE	-0.01	0.00	0.961	0.961	0.000
Motor	motor.LM1.Rhand.beta.rebound	ButtonR_LM1_to_LIFG_ΔPTE	-0.17	1.43	0.237	0.316	0.031
Motor	motor.RM1.Rhand.beta.rebound	ButtonR_RM1_to_RIFG_ΔPTE	-0.23	2.52	0.119	0.316	0.053
Auditory	audio.LA1s.beta.early	sound_LA1_to_LIFG_ΔPTE	0.12	0.71	0.402	0.608	0.016
Auditory	audio.RA1s.beta.early	sound_RA1_to_RIFG_ΔPTE	-0.08	0.27	0.608	0.608	0.006
Visual	visual.LV1s.beta.early	video_LV1_to_LIFG_ΔPTE	0.04	0.06	0.803	0.803	0.001
Visual	visual.RV1s.beta.early	video_RV1_to_RIFG_ΔPTE	0.12	0.79	0.379	0.758	0.017

Note. None of the reverse target→IFG models reached nominal significance or survived FDR correction.

Table S5. Directionality-index PTE models predicting target beta modulation.
Directionality-index models used IFG→target PTE minus target→IFG PTE as the predictor. FDR correction was applied across the 12 target-aligned tests. Eight of 12 directionality-index models survived FDR correction, paralleling the forward IFG→target pattern.

Domain	Target beta feature	PTE predictor	β	F(1,45)	p	qFDR	partial R^2
Motor	motor.LM1.Lhand.beta.early	ButtonL_LIFG_to_LM1_ΔPTE_dirIndex	-0.39	8.19	0.006	0.008	0.154
Motor	motor.RM1.Lhand.beta.early	ButtonL_RIFG_to_RM1_ΔPTE_dirIndex	-0.36	6.60	0.014	0.014	0.128
Motor	motor.LM1.Lhand.beta.rebound	ButtonL_LIFG_to_LM1_ΔPTE_dirIndex	-0.17	1.61	0.211	0.211	0.035
Motor	motor.RM1.Lhand.beta.rebound	ButtonL_RIFG_to_RM1_ΔPTE_dirIndex	-0.19	2.16	0.149	0.211	0.046
Motor	motor.LM1.Rhand.beta.early	ButtonR_LIFG_to_LM1_ΔPTE_dirIndex	-0.39	8.25	0.006	0.008	0.155
Motor	motor.RM1.Rhand.beta.early	ButtonR_RIFG_to_RM1_ΔPTE_dirIndex	-0.58	22.31	<0.001	<0.001	0.331
Motor	motor.LM1.Rhand.beta.rebound	ButtonR_LIFG_to_LM1_ΔPTE_dirIndex	-0.18	1.73	0.195	0.211	0.037

Motor	motor.RM1.Rhand.beta.rebound	ButtonR_RIFG_to_RM1_ΔPTE_dirIndex	-0.36	6.64	0.013	0.053	0.129
Auditory	audio.LA1s.beta.early	sound_LIFG_to_LA1_ΔPTE_dirIndex	-0.39	8.30	0.006	0.012	0.156
Auditory	audio.RA1s.beta.early	sound_RIFG_to_RA1_ΔPTE_dirIndex	-0.04	0.07	0.792	0.792	0.002
Visual	visual.LV1s.beta.early	video_LIFG_to_LV1_ΔPTE_dirIndex	-0.39	6.85	0.012	0.012	0.132
Visual	visual.RV1s.beta.early	video_RIFG_to_RV1_ΔPTE_dirIndex	-0.42	9.26	0.004	0.008	0.171

Note. Directionality-index models showed 7 of 12 associations surviving FDR correction, paralleling the forward IFG→target pattern with one M1 PMBR association falling below corrected significance.

Table S6. Clinical measures and their loadings on the clinical PC

**Clinical scores**	**Clinical measure**	**1st component loadings**
SOFAS	Social and Occupational Functioning Assessment Scale	**-.34**
PANSS-P1	Delusion	**.36**
PANSS-P2	Conceptual disorganization	.26
PANSS-P3	Hallucinatory Behavior	**.30**
PANSS-N1	Blunted affect	**.33**
PANSS-N4	Passive/apathetic social withdrawal	**.37**
PANSS-N6	Lack of spontaneity and flow of conversation	.17
PANSS-G5	Mannerisms/Posturing	.17
PANSS-G9	Unusual thought content	**.36**
CGI-S	Considering your total clinical experience with patients with schizophrenia, how severely ill has the patient been during the course of illness?	**.40**

Note: Feature loadings above 0.3 contribution to the component are bolded.

Table S7. Patient-only exploratory clinical correlations with behavioral, speech, and beta-summary metrics.

**Clinical**	**Metric**	**Pearson *r***	**Pearson *p***	**Pearson *q*FDR**	**Spearman ρ**	**Spearman *p***	**Spearman *q*FDR**
Illness-burden PC	SRR_A	0.04	0.861	0.952	0.09	0.676	0.929
	SRR_V	0.00	0.999	0.999	-0.05	0.813	0.929
	Pooled SRR	0.02	0.919	0.964	0.06	0.779	0.929
	Semantic PC	0.20	0.356	0.748	0.19	0.394	0.753
	Syntactic PC	0.40	0.059	0.308	0.42	0.045	0.281
	Semantic-beta latent score	-0.34	0.113	0.396	-0.31	0.146	0.341
	Syntactic-beta latent score	0.36	0.089	0.376	0.36	0.094	0.281
	SRR_A	-0.15	0.502	0.809	-0.02	0.929	0.929

Positive-symptom component	SRR_V	-0.17	0.427	0.805	-0.17	0.450	0.787
	Pooled SRR	-0.16	0.460	0.805	-0.09	0.670	0.929
	Semantic PC	0.25	0.254	0.592	0.34	0.113	0.297
	Syntactic PC	0.50	**0.014**	0.304	0.49	0.018	0.281
	Semantic-beta latent score	-0.41	0.053	0.308	-0.40	0.060	0.281
	Syntactic-beta latent score	0.45	**0.029**	0.308	0.45	0.031	0.281
Negative-symptom component	SRR_A	0.06	0.771	0.952	0.03	0.890	0.929
	SRR_V	0.04	0.843	0.952	0.03	0.887	0.929
	Pooled SRR	0.06	0.798	0.952	0.08	0.730	0.929
	Semantic PC	0.09	0.681	0.952	0.07	0.750	0.929
	Syntactic PC	0.13	0.540	0.809	0.25	0.259	0.543
	Semantic-beta latent score	-0.31	0.150	0.450	-0.38	0.078	0.281
	Syntactic-beta latent score	0.29	0.177	0.465	0.37	0.085	0.281

Note. Correlations were computed in patients only (*n* = 23). Clinical components included the illness-burden component reported in the main text, a positive-symptom component, and a negative-symptom component. Summary metrics included SRR_A, SRR_V, pooled SRR, semantic PC, syntactic PC, semantic-beta latent score, and syntactic-beta latent score. Pearson correlations were the primary exploratory analyses. Spearman correlations were included as sensitivity analyses because SRR measures were non-normally distributed. Benjamini-Hochberg false-discovery-rate correction was applied across the 21 tests in the Pearson matrix and separately across the 21 tests in the Spearman matrix. Uncorrected significance p-values are bolded. No association survived FDR correction.

REFERENCES

1. A. K. Engel, P. Fries, Beta-band oscillations—Signalling the status quo? *Curr. Opin. Neurobiol.* **20**, 156–165 (2010).

2. A. M. Bastos, W. M. Usrey, R. A. Adams, G. R. Mangun, P. Fries, K. J. Friston, Canonical microcircuits for predictive coding. *Neuron* **76**, 695–711 (2012).

3. B. Spitzer, S. Haegens, Beyond the status quo: A role for beta oscillations in endogenous content (re)activation. *eNeuro* **4**, 10.1523/ENEURO.0170-17.2017 (2017).

4. M. T. Wallace, R. A. Stevenson, The construct of the multisensory temporal binding window and its dysregulation in developmental disabilities. *Neuropsychologia* **64**, 105–123 (2014).

5. J.-P. Noel, R. A. Stevenson, M. T. Wallace, Atypical audiovisual temporal function in autism and schizophrenia: Similar phenotype, different cause. *Eur. J. Neurosci.* **47**, 1230–1241 (2018).

6. H. Zhou, X. Cai, M. Weigl, P. Bang, E. F. C. Cheung, R. C. K. Chan, Multisensory temporal binding window in autism spectrum disorders and schizophrenia spectrum disorders: A systematic review and meta-analysis. *Neurosci. Biobehav. Rev.* **86**, 66–76 (2018).

7. H. Zhou, I. Y. S. Lai, K. S. Y. Hung, M. K. M. Chan, Z. T. Y. Ho, J. P. H. Lam, S. S. Y. Lui, R. C. K. Chan, Audiovisual temporal processing in adult patients with first-episode schizophrenia and high-functioning autism. *Schizophrenia* **8**, 75 (2022).

8. M. T. Wallace, T. G. Woynaroski, R. A. Stevenson, Multisensory integration as a window into orderly and disrupted cognition and communication. *Annu. Rev. Psychol.* **71**, 193–219 (2020).

9. A. R. Powers III, A. R. Hillock, M. T. Wallace, Perceptual training narrows the temporal window of multisensory binding. *J. Neurosci.* **29**, 12265–12274 (2009).

10. M. Zerr, C. Freihorst, H. Schütz, C. Sinke, A. Müller, S. Bleich, T. F. Münte, G. R. Szycik, Brief sensory training narrows the temporal binding window and enhances long-term multimodal speech perception. *Front. Psychol.* **10**, 2489 (2019).

11. R. A. Stevenson, M. T. Wallace, Multisensory temporal integration: Task and stimulus dependencies. *Exp. Brain Res.* **227**, 249–261 (2013).

12. J.-P. Noel, K. J. Lakshminarasimhan, H. Park, D. E. Angelaki, Increased variability but intact integration during visual navigation in autism spectrum disorder. *Proc. Natl. Acad. Sci. U.S.A.* **117**, 11158–11166 (2020).

13. K. J. Friston, K. E. Stephan, R. Montague, R. J. Dolan, Computational psychiatry: The brain as a phantastic organ. *Lancet Psychiatry* **1**, 148–158 (2014).

14. R. A. Adams, K. E. Stephan, H. R. Brown, C. D. Frith, K. J. Friston, The computational anatomy of psychosis. *Front. Psych.* **4**, 47 (2013).

15. P. C. Fletcher, C. D. Frith, Perceiving is believing: A Bayesian approach to explaining the positive symptoms of schizophrenia. *Nat. Rev. Neurosci.* **10**, 48–58 (2009).

16. P. Sterzer, R. A. Adams, P. Fletcher, C. Frith, S. M. Lawrie, L. Muckli, P. Petrovic, P. Uhlhaas, M. Voss, P. R. Corlett, The predictive coding account of psychosis. *Biol. Psychiatry* **84**, 634–643 (2018).

17. P. R. Corlett, G. Horga, P. C. Fletcher, B. Alderson-Day, K. Schmack, A. R. Powers III, Hallucinations and strong priors. *Trends Cogn. Sci.* **23**, 114–127 (2019).

18. C. McNorgan, J. R. Booth, Skill dependent audiovisual integration in the fusiform induces repetition suppression. *Brain Lang.* **141**, 110–123 (2015).

19. B. M. Adhikari, E. S. Goshorn, B. Lamichhane, M. Dhamala, Temporal-order judgment of audiovisual events involves network activity between parietal and prefrontal cortices. *Brain Connect.* **3**, 536–545 (2013).

20. M. Dhamala, C. G. Assisi, V. K. Jirsa, F. L. Steinberg, J. A. S. Kelso, Multisensory integration for timing engages different brain networks. *Neuroimage* **34**, 764–773 (2007).

21. W. Sedley, P. E. Gander, S. Kumar, C. K. Kovach, H. Oya, H. Kawasaki, M. A. Howard III, T. D. Griffiths, Neural signatures of perceptual inference. *eLife* **5**, e11476 (2016).

22. L. H. Arnal, V. Wyart, A.-L. Giraud, Transitions in neural oscillations reflect prediction errors generated in audiovisual speech. *Nat. Neurosci.* **14**, 797–801 (2011).

23. A. R. Aron, S. Durston, D. M. Eagle, G. D. Logan, C. M. Stinear, V. Stuphorn, Converging evidence for a fronto-basal-ganglia network for inhibitory control of action and cognition. *J. Neurosci.* **27**, 11860–11864 (2007).

24. J. R. Wessel, A. R. Aron, On the globality of motor suppression: Unexpected events and their influence on behavior and cognition. *Neuron* **93**, 259–280 (2017).

25. A. R. Aron, T. W. Robbins, R. A. Poldrack, Right inferior frontal cortex: Addressing the rebuttals. *Front. Hum. Neurosci.* **8**, 905 (2014).

26. A. R. Aron, T. W. Robbins, R. A. Poldrack, Inhibition and the right inferior frontal cortex. *Trends Cogn. Sci.* **8**, 170–177 (2004).

27. G. A. Calvert, R. Campbell, M. J. Brammer, Evidence from functional magnetic resonance imaging of crossmodal binding in the human heteromodal cortex. *Curr. Biol.* **10**, 649–657 (2000).

28. V. Ojanen, R. Möttönen, J. Pekkola, I. P. Jääskeläinen, R. Joensuu, T. Autti, M. Sams, Processing of audiovisual speech in Broca's area. *Neuroimage* **25**, 333–338 (2005).

29. C. McGettigan, A. Faulkner, I. Altarelli, J. Obleser, H. Baverstock, S. K. Scott, Speech comprehension aided by multiple modalities: Behavioural and neural interactions. *Neuropsychologia* **50**, 762–776 (2012).

30. J. Hocking, C. J. Price, The influence of colour and sound on neuronal activation during visual object naming. *Brain Res.* **1241**, 92–102 (2008).

31. B. Straube, A. Green, S. Weis, T. Kircher, A supramodal neural network for speech and gesture semantics: An fMRI study. *PLOS ONE* **7**, e51207 (2012).

32. S. L. Thompson-Schill, M. D'Esposito, G. K. Aguirre, M. J. Farah, Role of left inferior prefrontal cortex in retrieval of semantic knowledge: A reevaluation. *Proc. Natl. Acad. Sci. U.S.A.* **94**, 14792–14797 (1997).

33. H. R. Snyder, M. T. Banich, Y. Munakata, Choosing our words: Retrieval and selection processes recruit shared neural substrates in left ventrolateral prefrontal cortex. *J. Cogn. Neurosci.* **23**, 3470–3482 (2011).

34. P. Indefrey, W. J. Levelt, The spatial and temporal signatures of word production components. *Cognition* **92**, 101–144 (2004).

35. P. Hagoort, On Broca, brain, and binding: A new framework. *Trends Cogn. Sci.* **9**, 416–423 (2005).

36. W. Matchin, E. Wood, Syntax-sensitive regions of the posterior inferior frontal gyrus and the posterior temporal lobe are differentially recruited by production and perception. *Cereb. Cortex Commun.* **1**, tgaa029 (2020).

37. A. D. Friederici, The brain basis of language processing: From structure to function. *Physiol. Rev.* **91**, 1357–1392 (2011).

38. A. G. Lewis, M. Bastiaansen, A predictive coding framework for rapid neural dynamics during sentence-level language comprehension. *Cortex* **68**, 155–168 (2015).

39. L. H. Arnal, A.-L. Giraud, Cortical oscillations and sensory predictions. *Trends Cogn. Sci.* **16**, 390–398 (2012).

40. M. F. Alonso-Sánchez, R. Limongi, J. Gati, L. Palaniyappan, Language network self-inhibition and semantic similarity in first-episode schizophrenia: A computational-linguistic and effective connectivity approach. *Schizophr. Res.* **259**, 97–103 (2023).

41. M. F. Alonso-Sánchez, W. Hinzen, R. He, J. Gati, L. Palaniyappan, Perplexity of utterances in untreated first-episode psychosis: An ultra–high field MRI dynamic causal modelling study of the semantic network. *J. Psychiatry Neurosci.* **49**, E252–E262 (2024).

42. D. Elleuch, Y. Chen, Q. Luo, L. Palaniyappan, Relationship between grammar and schizophrenia: A systematic review and meta-analysis. *Commun. Med.* **5**, 235 (2025).

43. K. Schneider, K. Leinweber, H. Jamalabadi, L. Teutenberg, K. Brosch, J.-K. Pfarr, F. Thomas-Odenthal, P. Usemann, A. Wroblewski, B. Straube, N. Alexander, I. Nenadić, A. Jansen, A. Krug, U. Dannlowski, T. Kircher, A. Nagels, F. Stein, Syntactic complexity and diversity of spontaneous speech production in schizophrenia spectrum and major depressive disorders. *Schizophrenia* **9**, 35 (2023).

44. M. F. Alonso-Sánchez, S. D. Ford, M. MacKinley, A. Silva, R. Limongi, L. Palaniyappan, Progressive changes in descriptive discourse in first episode schizophrenia: A longitudinal computational semantics study. *Schizophrenia* **8**, 36 (2022).

45. A. D. Friederici, C. J. Fiebach, M. Schlesewsky, I. D. Bornkessel, D. Y. von Cramon, Processing linguistic complexity and grammaticality in the left frontal cortex. *Cereb. Cortex* **16**, 1709–1717 (2005).

46. G. Pfurtscheller, F. H. Lopes da Silva, Event-related EEG/MEG synchronization and desynchronization: Basic principles. *Clin. Neurophysiol.* **110**, 1842–1857 (1999).

47. F. van Ede, S. Szebenyi, E. Maris, Attentional modulations of somatosensory alpha, beta and gamma oscillations dissociate between anticipation and stimulus processing. *Neuroimage* **97**, 134–141 (2014).

48. S. H. McGill, Q. Xin, T. Yadav, C. W. Zhao, P. Paszkowski, F. Darby, M. Guha, T. Nguyen, D. S. Jin, Y. Nir, J. Liu, L.-A. Sieu, H. Blumenfeld, Auditory sensory processing induces cortical and thalamic event-related desynchronization in the mouse. *PLOS ONE* **20**, e0334293 (2025).

49. C. E. Palmer, R. Auksztulewicz, S. Ondobaka, J. M. Kilner, Sensorimotor beta power reflects the precision-weighting afforded to sensory prediction errors. *Neuroimage* **200**, 59–71 (2019).

50. L. H. Arnal, Predicting "when" using the motor system's beta-band oscillations. *Front. Hum. Neurosci.* **6**, 225 (2012).

51. E. Heinrichs-Graham, M. J. Kurz, J. E. Gehringer, T. W. Wilson, The functional role of post-movement beta oscillations in motor termination. *Brain Struct. Funct.* **222**, 3075–3086 (2017).

52. A. A. Vakhtin, P. W. Kodituwakku, C. M. Garcia, C. D. Tesche, Aberrant development of post-movement beta rebound in adolescents and young adults with fetal alcohol spectrum disorders. *Neuroimage Clin.* **9**, 392–400 (2015).

53. H. Tan, C. Wade, P. Brown, Post-movement beta activity in sensorimotor cortex indexes confidence in the estimations from internal models. *J. Neurosci.* **36**, 1516–1528 (2016).

54. G. Hickok, Computational neuroanatomy of speech production. *Nat. Rev. Neurosci.* **13**, 135–145 (2012).

55. J. F. Houde, S. S. Nagarajan, Speech production as state feedback control. *Front. Hum. Neurosci.* **5**, 82 (2011).

56. J. R. Foucher, M. Lacambre, B.-T. Pham, A. Giersch, M. A. Elliott, Low time resolution in schizophrenia lengthened windows of simultaneity for visual, auditory and bimodal stimuli. *Schizophr. Res.* **97**, 118–127 (2007).

57. L. Gijbels, K. Lalonde, Y. Shen, M. T. Wallace, A. K. Lee, Synchrony perception of audiovisual speech is a reliable, yet individual construct. *Sci. Rep.* **15**, 15909 (2025).

58. P. F. Liddle, E. T. C. Ngan, S. L. Caissie, C. M. Anderson, A. T. Bates, D. J. Quested, R. White, R. Weg, Thought and Language Index: an instrument for assessing thought and language in schizophrenia. *Br J Psychiatry* **181**, 326–330 (2002).

59. N. C. Andreasen, Thought, language, and communication disorders. I. Clinical assessment, definition of terms, and evaluation of their reliability. *Arch Gen Psychiatry* **36**, 1315–1321 (1979).

60. G. Michalareas, J. Vezoli, S. van Pelt, J. M. Schoffelen, H. Kennedy, P. Fries, Alpha-beta and gamma rhythms subserve feedback and feedforward influences among human visual cortical areas. *Neuron* **89**, 384–397 (2016).

61. S. van Pelt, L. Heil, J. Kwisthout, S. Ondobaka, I. van Rooij, H. Bekkering, Beta- and gamma-band activity reflect predictive coding in the processing of causal events. *Soc. Cogn. Affect. Neurosci.* **11**, 973–980 (2016).

62. P. J. Uhlhaas, W. Singer, Abnormal neural oscillations and synchrony in schizophrenia. *Nat. Rev. Neurosci.* **11**, 100–113 (2010).

63. L. Sun, N. Castellanos, C. Grützner, D. Koethe, D. Rivolta, M. Wibral, L. Kranaster, W. Singer, M. F. Leweke, P. J. Uhlhaas, Evidence for dysregulated high-frequency oscillations during sensory processing in medication-naïve, first episode schizophrenia. *Schizophr. Res.* **150**, 519–525 (2013).

64. G. Northoff, J. Gomez-Pilar, Overcoming rest–task divide—Abnormal temporospatial dynamics and its cognition in schizophrenia. *Schizophr. Bull.* **47**, 751–765 (2021).

65. J. J. Newson, T. C. Thiagarajan, EEG frequency bands in psychiatric disorders: A review of resting state studies. *Front. Hum. Neurosci.* **12**, 521 (2019).

66. S. P. Ahlfors, J. Han, J. W. Belliveau, M. S. Hämäläinen, Sensitivity of MEG and EEG to source orientation. *Brain Topogr.* **23**, 227–232 (2010).

67. J. Vorwerk, Ü. Aydin, C. H. Wolters, C. R. Butson, Influence of head tissue conductivity uncertainties on EEG dipole reconstruction. *Front. Neurosci.* **13**, 531 (2019).

68. M. C. Piastra, A. Nüßing, J. Vorwerk, M. Clerc, C. Engwer, C. H. Wolters, A comprehensive study on electroencephalography and magnetoencephalography sensitivity to cortical and subcortical sources. *Hum. Brain Mapp.* **42**, 978–992 (2020).

69. I. Goodwin, K. M. J. Diederen, E. J. Hird, V. Weilnhammer, M. I. Garrido, F. Knolle, Predictive processing accounts of psychosis: Bottom-up or top-down disruptions. *Nat. Ment. Health* **4**, 60–84 (2026).

70. A. Wolff, J. Gomez-Pilar, J. Zhang, J. Choueiry, S. de la Salle, V. Knott, G. Northoff, It's in the timing: Reduced temporal precision in neural activity of schizophrenia. *Cereb. Cortex* **32**, 3441–3456 (2022).

71. A. Wolff, G. Northoff, Temporal imprecision of phase coherence in schizophrenia and psychosis-dynamic mechanisms and diagnostic marker. *Mol. Psychiatry* **29**, 425–438 (2024).

72. S. Lechner, G. Northoff, Temporal imprecision and phase instability in schizophrenia resting state EEG. *Asian J. Psychiatr.* **86**, 103654 (2023).

73. S. Lechner, M. H. Hsieh, Y.-T. Lin, C.-M. Liu, I.-F. Chen, C.-C. Liu, Y.-L. Chien, T.-J. Hwang, H.-G. Hwu, G. Northoff, Temporal imprecision and its dynamics in schizophrenia. *Transl. Psychiatry* **15**, 279 (2025).

74. T. T. J. Kircher, T. M. Oh, M. J. Brammer, P. K. McGuire, Neural correlates of syntax production in schizophrenia. *Br. J. Psychiatry* **186**, 209–214 (2005).

75. L. Palaniyappan, N. B. Mota, S. Oowise, V. Balain, M. Copelli, S. Ribeiro, P. F. Liddle, Speech structure links the neural and socio-behavioural correlates of psychotic disorders. *Prog. Neuropsychopharmacol. Biol. Psychiatry* **88**, 112–120 (2019).

76. A. G. Lewis, J.-M. Schoffelen, H. Schriefers, M. Bastiaansen, A predictive coding perspective on beta oscillations during sentence-level language comprehension. *Front. Hum. Neurosci.* **10**, 85 (2016).

77. C. G. Richter, R. Coppola, S. L. Bressler, Top-down beta oscillatory signaling conveys behavioral context in early visual cortex. *Sci. Rep.* **8**, 6991 (2018).

78. E. Tang, N. Chen, J. Li, Y. Liu, H. Ding, H. Chen, Neurobehavioral characteristics and symptomatic correlations of audiovisual multisensory integration in schizophrenia: A systematic review and meta-analysis. *J. Psychiatr. Res.* **185**, 11–25 (2025).

79. B. Habelt, The potential of event-related beta oscillations as biomarkers for neuromodulatory treatment efficacy. *Front. Psychol.* **17**, 1779055 (2026).

80. P. L. Morosini, L. Magliano, L. Brambilla, S. Ugolini, R. Pioli, Development, reliability and acceptability of a new version of the DSM-IV Social and Occupational Functioning Assessment Scale (SOFAS) to assess routine social functioning. *Acta Psychiatr Scand* **101**, 323–329 (2000).

81. C.-H. Lin, H.-S. Lin, S.-C. Lin, C.-C. Kuo, F.-C. Wang, Y.-H. Huang, Early improvement in PANSS-30, PANSS-8, and PANSS-6 scores predicts ultimate response and remission during acute treatment of schizophrenia. *Acta Psychiatr Scand* **137**, 98–108 (2018).

82. J. M. Haro, S. A. Kamath, S. Ochoa, D. Novick, K. Rele, A. Fargas, M. J. Rodríguez, R. Rele, J. Orta, A. Kharbeng, S. Araya, M. Gervin, J. Alonso, V. Mavreas, E. Lavrentzou, N. Liontos, K. Gregor, P. B. Jones, SOHO Study Group, The Clinical Global Impression-Schizophrenia scale: a simple instrument to measure the diversity of symptoms present in schizophrenia. *Acta Psychiatr Scand Suppl*, 16–23 (2003).

83. L. Palaniyappan, M. F. Alonso-Sanchez, V. Bambini, E. Bora, W. Hinzen, G. Kuperberg, B. MacWhinney, N. Mota, J. Olah, S. Rossell, I. E. Sommer, DISCOURSE in Psychosis: introducing an international consortium for research on thought, language and communication. *World Psychiatry*. **25**, 490–492 (2026).

84. B. D. Van Veen, W. van Drongelen, M. Yuchtman, A. Suzuki, Localization of brain electrical activity via linearly constrained minimum variance spatial filtering. *I.E.E.E. Trans. Biomed. Eng.* **44**, 867–880 (1997).

85. A. Hillebrand, G. R. Barnes, Beamformer analysis of MEG data. *Int. Rev. Neurobiol.* **68**, 149–171 (2005).

86. C. Destrieux, B. Fischl, A. Dale, E. Halgren, Automatic parcellation of human cortical gyri and sulci using standard anatomical nomenclature. *Neuroimage* **53**, 1–15 (2010).

87. C. Pantev, M. Hoke, K. Lehnertz, B. Lütkenhöner, G. Anogianakis, W. Wittkowski, Tonotopic organization of the human auditory cortex revealed by transient auditory evoked magnetic fields. *Electroencephalogr. Clin. Neurophysiol.* **69**, 160–170 (1988).

88. L. Lehtelä, R. Salmelin, R. Hari, Evidence for reactive magnetic 10-Hz rhythm in the human auditory cortex. *Neurosci. Lett.* **222**, 111–114 (1997).

89. T. Fujioka, B. Ross, L. J. Trainor, Beta-band oscillations represent auditory beat and its metrical hierarchy in perception and imagery. *J. Neurosci.* **35**, 15187–15198 (2015).

90. S. Hanslmayr, W. Klimesch, P. Sauseng, W. Gruber, M. Doppelmayr, R. Freunberger, T. Pecherstorfer, Visual discrimination performance is related to decreased alpha amplitude but increased phase locking. *Neurosci. Lett.* **375**, 64–68 (2005).

91. C. Tallon-Baudry, O. Bertrand, C. Delpuech, J. Pernier, Oscillatory γ-band (30–70 Hz) activity induced by a visual search task in humans. *J. Neurosci.* **17**, 722–734 (1997).

92. M. Binder, Neural correlates of audiovisual temporal processing—Comparison of temporal order and simultaneity judgments. *Neuroscience* **300**, 432–447 (2015).

93. K. O. Bushara, J. Grafman, M. Hallett, Neural correlates of auditory–visual stimulus onset asynchrony detection. *J. Neurosci.* **21**, 300–304 (2001).

94. M. T. Jurkiewicz, W. C. Gaetz, A. C. Bostan, D. Cheyne, Post-movement beta rebound is generated in motor cortex: Evidence from neuromagnetic recordings. *Neuroimage* **32**, 1281–1289 (2006).

95. B. E. Kilavik, M. Zaepffel, A. Brovelli, W. A. MacKay, A. Riehle, The ups and downs of β oscillations in sensorimotor cortex. *Exp. Neurol.* **245**, 15–26 (2012).

96. N. C. Swann, W. Cai, C. R. Conner, T. A. Pieters, M. P. Claffey, J. S. George, A. R. Aron, N. Tandon, Roles for the pre-supplementary motor area and the right inferior frontal gyrus in stopping action: Electrophysiological responses and functional and structural connectivity. *Neuroimage* **59**, 2860–2870 (2012).

97. T. Lennert, S. Samiee, S. Baillet, Coupled oscillations enable rapid temporal recalibration to audiovisual asynchrony. *Commun. Biol.* **4**, 559 (2021).

98. T. D. Griffiths, J. D. Warren, What is an auditory object? *Nat. Rev. Neurosci.* **5**, 887–892 (2004).

99. R. Näätänen, T. Picton, The N1 wave of the human electric and magnetic response to sound: A review and an analysis of the component structure. *Psychophysiology* **24**, 375–425 (1987).

100. J. Sassenhagen, D. Draschkow, Cluster-based permutation tests of MEG/EEG data do not establish significance of effect latency or location. *Psychophysiology* **56**, e13335 (2019).

101. V. van Wassenhove, K. W. Grant, D. Poeppel, Temporal window of integration in auditory-visual speech perception. *Neuropsychologia* **45**, 598–607 (2007).

102. A. Todorovic, F. P. de Lange, Repetition suppression and expectation suppression are dissociable in time in early auditory evoked fields. *J. Neurosci.* **32**, 13389–13395 (2012).

103. L. H. Arnal, K. B. Doelling, D. Poeppel, Delta-beta coupled oscillations underlie temporal prediction accuracy. *Cereb. Cortex* **25**, 3077–3085 (2015).

104. G. Northoff, A. Koch, J. Wenke, J. Eckert, H. Böker, B. Pflug, B. Bogerts, Catatonia as a psychomotor syndrome: A rating scale and extrapyramidal motor symptoms. *Mov. Disord.* **14**, 404–416 (1999).

105. M. Lobier, F. Siebenhühner, S. Palva, J. M. Palva, Phase transfer entropy: A novel phase-based measure for directed connectivity in networks coupled by oscillatory interactions. *Neuroimage* **85** (Pt. 2), 853–872 (2014).
